# *N*‑Boryl Pyridyl Radical-Mediated Reductive Homocoupling of Arylsulfonyl Chlorides to Diaryl Disulfides

**DOI:** 10.1021/acs.joc.5c02723

**Published:** 2025-12-23

**Authors:** Yuan-Kai Cheng, Chien-Miao Li, Woo-Jin Yoo

**Affiliations:** † Department of Chemistry, 33561 National Taiwan University, No. 1, Sec. 4, Roosevelt Road, Taipei 10617, Taiwan; ‡ Center for Emerging Materials and Advanced Devices, National Taiwan University, No. 1, Sec. 4, Roosevelt Road, Taipei 10617, Taiwan

## Abstract

Arylsulfonyl chlorides were found to undergo 4-cyanopyridine-catalyzed, bis­(pinacolato)­diboron-mediated reductive homocoupling to afford a variety of symmetrical diaryl disulfides. Preliminary mechanistic studies suggest that arylsulfonyl radicals are involved and that diaryl disulfide formation likely proceeds through successive single-electron reductions mediated by *N*-boryl pyridyl radicals.

## Graphical abstract

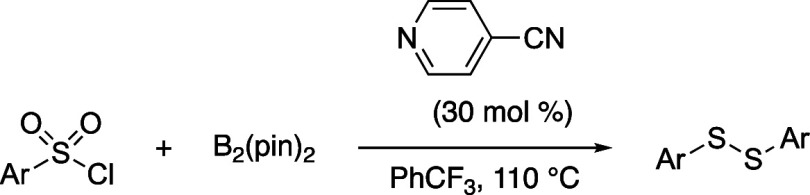

Diboron­(4) compounds are a versatile class of reductants and boron-transfer reagents that are widely employed in organic synthesis.^1^ These reagents are typically activated by transition metal complexes, nucleophilic organocatalysts, or strong Brønsted bases to facilitate diverse borylation and reduction reactions. More recently, pyridine derivatives have been shown to cleave the B–B bond of tetraalkoxydiboron(4) to generate reactive boryl radicals and anionic intermediates that function as single-electron reductants and as sources of both boryl and pyridyl radicals to enable a range of radical-mediated transformations under metal-free conditions (Scheme 1a).^2^ One of the earliest applications of these intermediates was their use to facilitate dehalogenative C-borylation of aryl halides to form aryl boronic esters.^3a^ Since then, several different aryl and aliphatic compounds with redox-active functional groups have been reported as viable substrates for borylation reactions (Scheme 1b).^3–6^

**Scheme 1:**
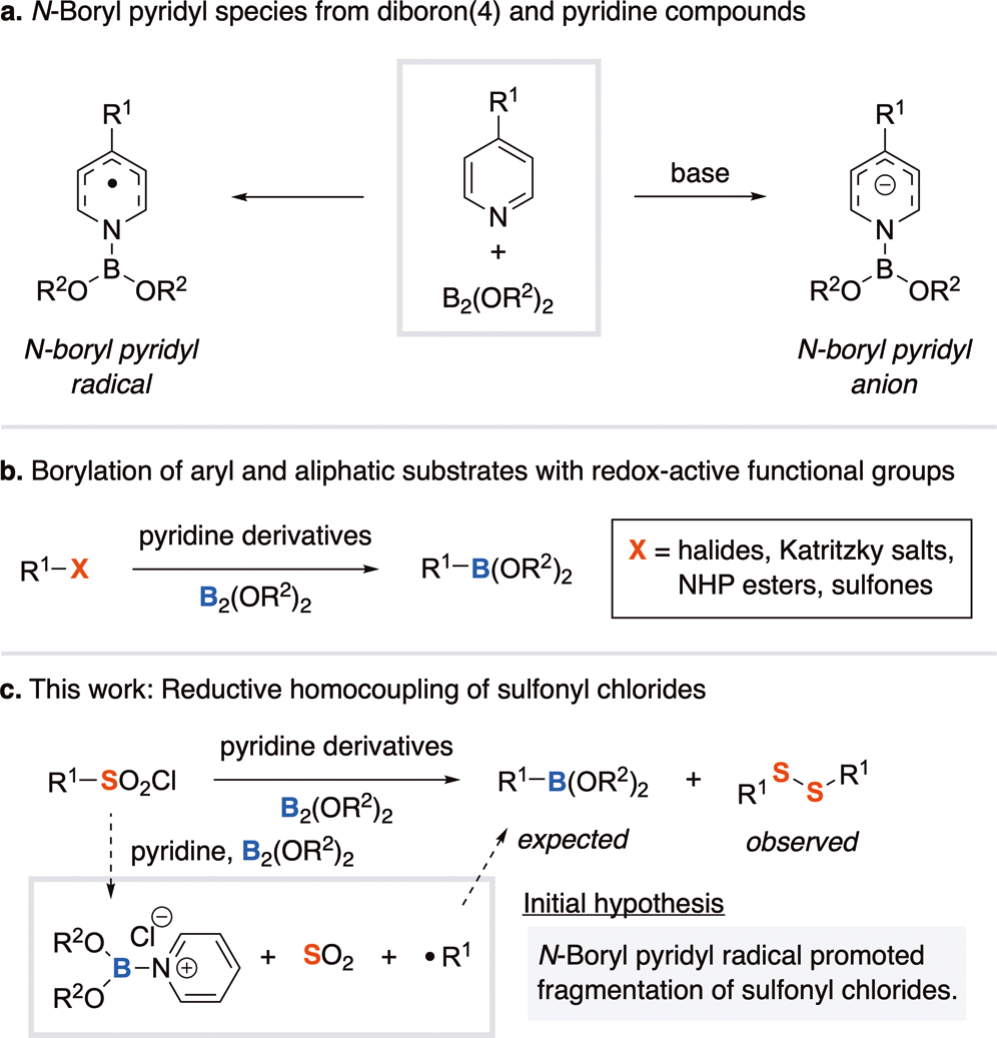
Activation of Diboron(4) Compounds by Pyridine Derivatives

Organosulfur compounds are widely represented among natural products and bioactive molecules.^7^ Sulfonyl chlorides, which are inexpensive and readily accessible electrophiles, are often employed to introduce sulfur-containing functional groups into organic frameworks. In recent years, sulfonyl chlorides have emerged as versatile radical precursors under visible light-induced photoredox conditions.^8^ While aryl sulfonyl chlorides typically generate sulfonyl radical intermediates, there are also reports in which reductive fragmentation leads to the formation of aryl radicals.^9^ We initially hypothesized that *N*-boryl pyridyl radicals/anions could promote the reductive cleavage of arylsulfonyl chlorides to generate aryl radicals, which could then be intercepted by a diboron(4) reagent to furnish aryl boronic esters. However, over the course of our investigations, the hypothesized aryl boronic ester was not detected, and a diaryl disulfide was obtained instead (Scheme 1c). While the reductive homocoupling of sulfonyl halides has been previously reported,^10^ to the best of our knowledge, the use of *N*-boryl pyridyl intermediates to mediate this transformation has not yet been described. Herein, we disclose a 4-cyanopyridine-catalyzed B_2_(pin)_2_-mediated reductive coupling of arylsulfonyl chlorides to afford a series of diaryl disulfides.

We began our studies by applying the reported conditions for the decarboxylative borylation of redox-active esters^4b^ to benzenesulfonyl chloride (**1a**) and observed a complex mixture with diphenyl disulfide (**3a**) as the major product (Table 1, entry 1). Doubling the catalyst loading of ethyl isonicotinate (**2a**) improved the yield of **3a** (entry 2), while screening various pyridine derivatives revealed 4-cyanopyridine (**2b**) as an effective catalyst for the reductive coupling process (entries 3–5). We examined various solvents (entries 6–8) and diboron(4) compounds (entries 9–10), but in all cases, they failed to improve the yield of **3a**.

**Table 1:**
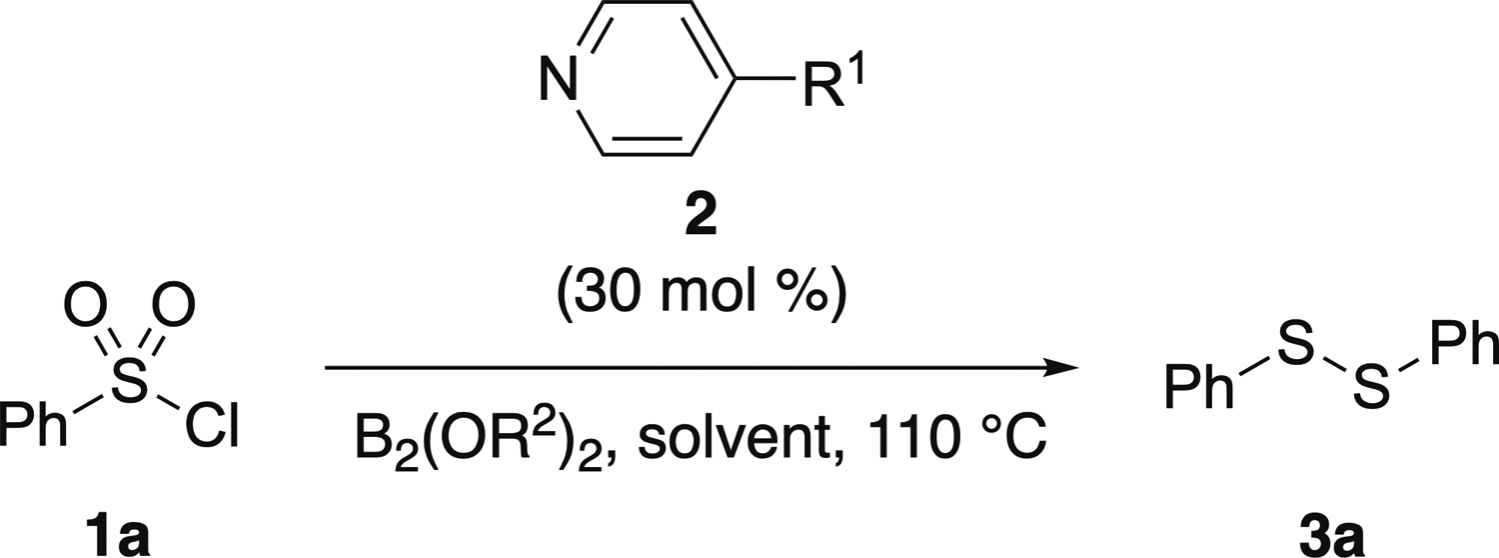
Optimization Studies for the Reductive Homocoupling of Phenylsulfonyl Chloride (**1a**)­^a^

entry	R^1^	B_2_(OR^2^)_2_	solvent	yield (%)^b^
1^c^	CO_2_Et (**2a**)	B_2_(pin)_2_	PhCF_3_	24
2	CO_2_Et (**2a**)	B_2_(pin)_2_	PhCF_3_	80
3	CN (**2b**)	B_2_(pin)_2_	PhCF_3_	87
4	Ph (**2c**)	B_2_(pin)_2_	PhCF_3_	N.D.
5	H (**2d**)	B_2_(pin)_2_	PhCF_3_	N.D.
6	CN (**2b**)	B_2_(pin)_2_	Toluene	70
7	CN (**2b**)	B_2_(pin)_2_	MeCN	63
8	CN (**2b**)	B_2_(pin)_2_	MTBE	83
9	CN (**2b**)	B_2_(nep)_2_	PhCF_3_	68
10	CN (**2b**)	B_2_(cat)_2_	PhCF_3_	45

a Reaction conditions: sulfonyl chloride **1a** (0.40 mmol), B_2_(OR^2^)_2_ (1.2 mmol), pyridine derivative **2** (0.12 mmol, 30 mol %), and solvent (2.0 mL) at 110 °C for 15 h under an atmosphere of N_2_.

b Yield based on **1a** and determined by ^1^H NMR analysis using 1,1,2,2-tetrachloroethane as an internal standard.

c Used 15 mol % of ethyl isonicotinate (**2a**).

With the optimized conditions in hand, the substrate scope of the 4-cyanopyridine-catalyzed reductive homocoupling of sulfonyl chlorides was explored (Table 2). A variety of aryl and polyaromatic sulfonyl chlorides (**1a**-**n**) were evaluated, generally affording good yields, although substrates bearing electron-withdrawing substituents tended to give slightly lower yields (entries 1–14). Notably, the reaction exhibited good functional group tolerance, accommodating a range of potentially reducible substituents, including halides, esters, and nitriles. However, exceptions were observed with 4-nitrobenzenesulfonyl chloride (**1j**) and 2-cyanobenzenesulfonyl chloride (**1m**), both of which led to the formation of a mixture of unidentified products.^11,12^ Sulfonyl chlorides bearing heteroaromatic substituents were also investigated (entries 15 and 16). Thiophene-2-sulfonyl chloride (**1o**) afforded the disulfide product **3o** in moderate yield, whereas pyridine-3- sulfonyl chloride (**1p**) failed to deliver the expected disulfide **3p**. Finally, an aliphatic sulfonyl chloride, such as benzyl sulfonyl chloride (**1q**), was examined as a substrate (entry 17). It was found that the reaction resulted in a complex mixture, with the desired dialkyl disulfide **3q** detected only in trace amounts.

**Table 2:**
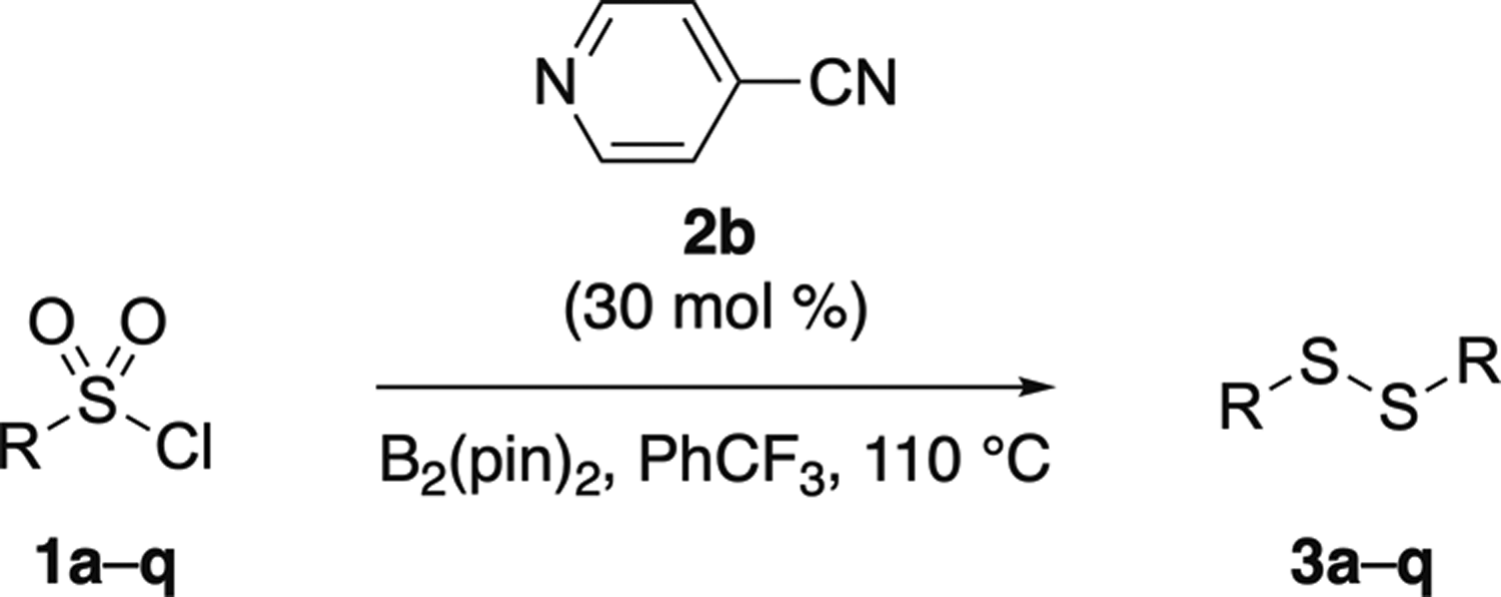
Substrate Scope for the Reductive Homocoupling of Sulfonyl Chlorides **1a–q** ^a^

entry	R	yield (%)^b^
1	Ph (**1a**)	99
2	4-Me-C_6_H_4_ (**1b**)	92
3	4-MeO-C_6_H_4_ (**1c**)	91
4	3-MeO-C_6_H_4_ (**1d**)	69
5	2-MeO-C_6_H_4_ (**1e**)	85
6	4-Ph-C_6_H_4_ (**1f**)	78
7	4-Cl-C_6_H_4_ (**1g**)	90
8	4-Br-C_6_H_4_ (**1h**)	72
9	4-CO_2_Me-C_6_H_4_ (**1i**)	78
10	4-NO_2_-C_6_H_4_ (**1j**)	N.D.
11	4-CN-C_6_H_4_ (**1k**)	81
12	3-CN-C_6_H_4_ (**1l**)	75
13	2-CN-C_6_H_4_ (**1m**)	N.D.
14	2-naphthyl (**1n**)	82
15	2-thiophenyl (**1o**)	42
16	3-pyridyl (**1p**)	N.D.
17	Bn (**1q**)	<5^c^

a Reaction conditions: sulfonyl chloride **1a**–**q** (0.40 mmol), B_2_(pin)_2_ (1.2 mmol), 4-cyanopyridine (**2b**) (0.12 mmol, 30 mol %), and PhCF_3_ (2.0 mL) at 110 °C for 15 h under an atmosphere of N_2_.

b Yields of isolated products **3a**–**o** based on **1a**–**o**.

c Yield of **3q** determined by ^1^H NMR analysis of the crude using 1,1,2,2-tetrachloroethane as an internal standard.

To probe the mechanism for the reductive homocoupling of aryl sulfonyl chlorides, a series of control experiments were conducted (Scheme 2). Since the activation of diboron(4) reagents by 4-cyanopyridine is known to generate *N*-boryl pyridyl radical intermediates,^13^ the model substrate **1a** was subjected to the optimized conditions in the presence of 2,2,6,6-tetramethylpiperidine-1-oxyl (TEMPO), butylated hydroxytoluene (BHT), and 1,1-diphenylethylene (DPE) as radical inhibitors (Scheme 2a). These inhibition experiments revealed that all three radical inhibitors effectively suppressed the reaction, supporting the involvement of a radical pathway in this reductive process. Moreover, high resolution mass spectrometry provided direct evidence for phenylsulfonyl radical formation by detecting the corresponding BHT and DPE adducts, respectively. We next turned our attention to identifying plausible intermediates involved in the reductive homocoupling process (Scheme 2b). According to literature reports, three prevailing mechanistic pathways have been proposed, involving either aryl sulfenyl halides,^10b,10h^ aryl sulfinates,^10i^ or diaryl disulfones^10c^ as intermediates. To identify the most plausible mechanism under our reaction conditions, potential intermediates **4**–**6** were subjected to an optimized protocol. Among them, only sodium benzenesulfinate (**5**) failed to produce diphenyl disulfide (**3a**). Since aryl sulfenyl halides have been proposed as intermediates in the reductive sulfenylation of indoles with arylsulfonyl chlorides,^14^ indole was introduced into the reaction system to probe the formation of benzenesulfenyl chloride (**4**). The formation of 3-sulfenylated indole **7** in good yield provides additional support for the intermediacy of **4** (Scheme 2c, eq 1). Regarding diaryl disulfones as intermediates, their conversion into diaryl disulfides would likely require multiple deoxygenative O-atom transfer steps. However, it was found that diphenyl sulfone (**8**) could not be reduced to diphenyl sulfide (**9**) under the optimized conditions (eq 2). A possible explanation for the observed reduction of diphenyl disulfone (**6**) is that phenylsulfonyl radical, generated from the homolytic cleavage of the S–S bond,^15,16^ may serve as an intermediate in the formation of diphenyl disulfide (**3a**).

**Scheme 2:**
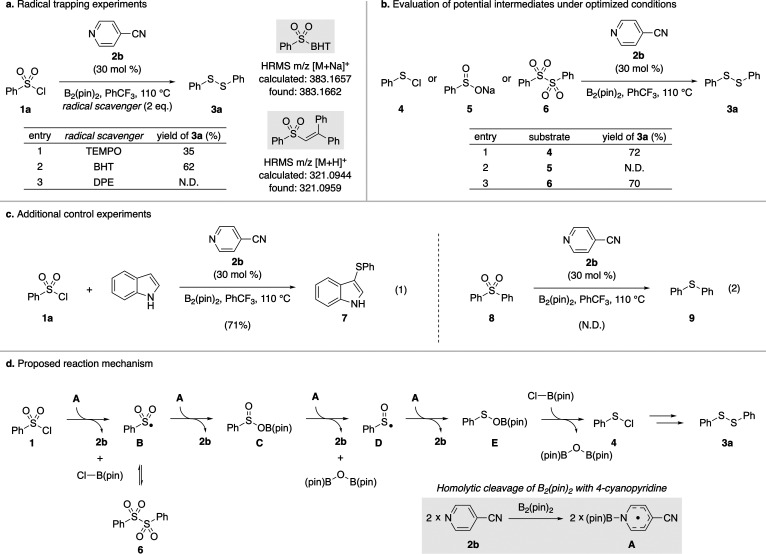
Preliminary Mechanistic Investigations

Based on previous reports and our experimental findings, we propose the mechanism depicted in Scheme 2d. It has been reported that B_2_(pin)_2_ can be homolytically cleaved by **2b** to generate *N*-boryl pyridyl radical **A**, which could engage with phenylsulfonyl chloride (**1a**) to yield sulfonyl radical **B**.^13^ The ensuing radical–radical coupling of **A** and **B** furnishes sulfinate intermediate **C**, which may undergo deoxygenation upon reaction with another equivalent of **A** to generate phenylsulfinyl radical **D**. Subsequent addition of *N*-boryl pyridyl radical **A** could convert **D** into **E**, which may then react with B-chloro-pinacolborane to form phenylsulfenyl chloride (**4**). Finally, the reductive homocoupling of **4** would produce diphenyl disulfide (**3a**).

To summarize, we have discovered a new application of *N*-boryl pyridyl radicals in organic synthesis, demonstrating their ability to mediate the reductive homocoupling of arylsulfonyl chlorides to form symmetrical diaryl disulfides. Preliminary mechanistic studies indicate that *N*-boryl pyridyl radicals most likely generate arylsulfonyl radicals, which, through successive O-atom transfers, result in the formation of diaryl disulfides. This work not only provides an alternative method for diaryl disulfide synthesis but also highlights the expanded utility of *N*-boryl pyridyl radicals in radical-mediated organic transformations.

## Supplementary Material



## Data Availability

The data underlying this study are available in the published article and its Supporting Information.

## Notes

Experimental procedures, characterization data, and copies of ^1^H and ^13^C NMR spectra (PDF)
