# Supplementary material for: N‑Boryl Pyridyl Radical-Mediated Reductive Homocoupling of Arylsulfonyl Chlorides to Diaryl Disulfides
Source: J Org Chem. 2025 Dec 23;91(1):810–4. doi: 10.1021/acs.joc.5c02723 (PMC12797280; doi:10.1021/acs.joc.5c02723)
Supplement: Supplementary file 1 [file jo5c02723_si_001.pdf]

Supporting Information

***N*-Boryl Pyridyl Radical Mediated Reductive Homocoupling of Arylsulfonyl Chlorides to Diaryl Disulfides**

Yuan-Kai Cheng,[a] Chien-Miao Li,[a] and Woo-Jin Yoo[a,b]*

[a] Department of Chemistry, National Taiwan University, No. 1, Sec. 4, Roosevelt Road, Taipei 10617, Taiwan

[b] Center for Emerging Materials and Advanced Devices, National Taiwan University, No. 1, Sec. 4, Roosevelt Road, Taipei 10617, Taiwan

Email: woojinyoo@ntu.edu.tw

Table of Contents

Part I: Substrate Scope for the Reductive Homocoupling of Sulfonyl Chlorides **1a-o**..........S-2

Part II: Radical Trapping Experiments with **1a**..........S-5

Part III: Examination of Potential Intermediates **4**–**6** under Optimized Conditions..........S-6

Part IV: Additional Control Experiments..........S-6

Part V: References..........S-7

Part VI: Copies of NMR Spectra Data..........S-9

General Information: ^{1}H, and ^{13}C, and spectra were recorded on Bruker AVIII HD 400 spectrometer in $CDCl_3$. Chemical shifts were reported in parts per million (ppm) from chloroform using the solvent resonance as the internal standard ($CHCl_3$: δ 7.26 ppm) or tetramethylsilane (δ 0.00 ppm) for ^{1}H NMR and ($CDCl_3$: δ 77.00 ppm) for ^{13}C NMR. Column chromatography was carried out using either silica gel (Silica gel 60, 40-63 μm) from Merck.

Reagents: Unless stated otherwise, commercial reagents (Energy Chemical, Nova Materials, Sigma-Aldrich, Thermo Fisher Scientific, Merck Millipore, Shimakyu Chemical, TCI, Tedia, Encore Chemicals, ECHO Chemical, Duksan Pure Chemicals, and Matrix Scientific) were used without purification. Sulfonyl chlorides **1a**–**q** were purchased from Energy Chemical and used as received. Solvents were either purified by distillation under dry nitrogen from CaH_2 or obtained from a solvent purification system (Vigor).

Part I: Substrate Scope for the Reductive Homocoupling of Sulfonyl Chlorides **1a-o**

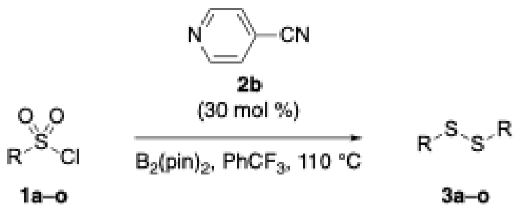

In a glovebox, an oven-dried vial equipped with a stir bar was charged with **1a-o** (0.400 mmol), $B_2(pin)_2$ (304.7 mg, 1.200 mmol), 4-cyanopyridine (**2b**) (12.5 mg, 0.120 mmol), and dried, degassed benzotrifluoride (2 mL). The vial was sealed with a Telfon-lined cap and stirred in an oil bath preheated to 110 °C for 15 h. After cooling to room temperature, the reaction mixture was directly purified by flash column chromatography to afford **3a-o**.

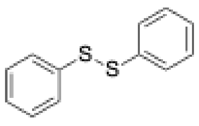

3a

1,2-Diphenyldisulfane (3a).[1] Following the above procedure with **1a** (70.6 mg, 0.400 mmol). The crude product was purified by flash column chromatography to afford **3a** as a white solid (43.3 mg, 99%). R_f = 2.7/4.0 (20% EtOAc in hexane); ^{1}H NMR (400 MHz, $CDCl_3$) δ 7.52-7.49 (m, 4H), 7.33-7.28 (m, 4H), 7.25-7.20 (m, 2H); $^{13}C\{^1H\}$ NMR ($CDCl_3$, 101 MHz) δ 131.2, 129.2, 127.7, 127.3.

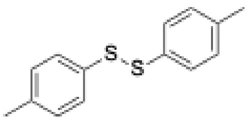

3b

1,2-Di-*p*-tolyldisulfane (3b).[2] Following the above procedure with **1b** (76.3 mg, 0.400 mmol). The crude product was purified by flash column chromatography to afford **3b** as a white solid (45.4 mg, 92%). R_f = 2.8/4.0 (20% EtOAc in hexane); ^{1}H NMR (400 MHz, $CDCl_3$) δ 7.40 (d, *J* = 8.2 Hz, 4H), 7.12 (d, *J* = 7.97 Hz, 4H), 2.34 (s, 6H); $^{13}C\{^1H\}$ NMR ($CDCl_3$, 101 MHz) δ 137.6, 134.1, 129.1, 128.7, 21.2.

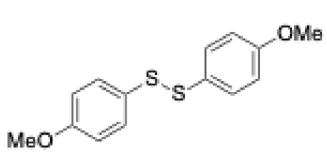

3c

1,2-Bis(4-methoxyphenyl)disulfane (3c).[2] Following the above procedure with **1c** (82.7 mg, 0.400 mmol). The crude product was purified by flash column chromatography to afford **3c** as a white solid (54.0 mg, 91%). R_f = 2.0/4.0 (20% EtOAc in hexane); ^{1}H NMR (400 MHz, $CDCl_3$) δ 7.43-7.38 (m, 4H), 6.86-6.82 (m, 4H), 3.80 (s, 6H); ^{13}C{^{1}H} NMR ($CDCl_3$, 101 MHz) δ 160.1, 132.8, 128.6, 114.8, 55.5.

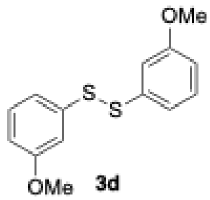

3d

1,2-Bis(3-methoxyphenyl)disulfane (3d).[3] Following the above procedure with **1d** (82.7 mg, 0.400 mmol). The crude product was purified by flash column chromatography to afford **3d** as a pale yellow oil (38.5 mg, 69%). R_f = 2.3/4.0 (20% EtOAc in hexane); ^{1}H NMR (400 MHz, $CDCl_3$) δ 7.23-7.20 (m, 2H), 7.10-7.07 (m, 4H), 6.79-6.75 (m, 2H), 3.77 (s, 6H); ^{13}C{^{1}H} NMR ($CDCl_3$, 101 MHz) δ 160.2, 138.4, 130.0, 119.8, 113.3, 112.8, 55.4.

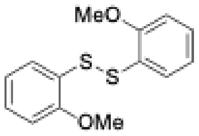

3e

1,2-Bis(2-methoxyphenyl)disulfane (3e).[4] Following the above procedure with **1e** (82.7 mg, 0.400 mmol). The crude product was purified by flash column chromatography to afford **3e** as a pale yellow solid (47.5 mg, 85%). R_f = 2.1/4.0 (20% EtOAc in hexane); ^{1}H NMR (400 MHz, $CDCl_3$) δ 7.55 (dd, J = 7.7, 1.5 Hz, 2H), 7.19 (td, J = 8.1, 1.5 Hz, 2H), 6.92 (td, J = 7.8, 1.0 Hz, 2H), 6.86 (d, J = 8.0 Hz, 2H), 3.90 (s, 6H); ^{13}C{^{1}H} NMR ($CDCl_3$, 101 MHz) δ 156.7, 127.9, 127.8, 124.7, 121.4, 110.7, 56.0.

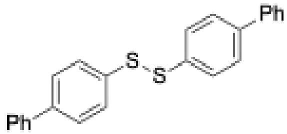

3f

1,2-Di([1,1'-biphenyl]-4-yl)disulfane (3f).[2] Following the above procedure with **1f** (101.1 mg, 0.4001 mmol). The crude product was purified by flash column chromatography to afford **3f** as a white solid (57.8 mg, 78%). R_f = 2.5/4.0 (20% EtOAc in hexane); ^{1}H NMR (400 MHz, $CDCl_3$) δ 7.65-7.54 (m, 12H), 7.48-7.42 (m, 4H), 7.39-7.33 (m, 2H); ^{13}C{^{1}H} NMR ($CDCl_3$, 101 MHz) δ 140.5, 140.3, 136.2, 129.0, 128.4, 127.9, 127.7, 127.1.

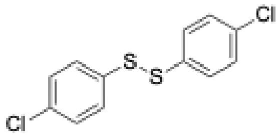

3g

1,2-Bis(4-chlorophenyl)disulfane (3g).[2] Following the above procedure with **1g** (84.4 mg, 0.400 mmol). The crude product was purified by flash column chromatography to afford **3g** as a pale yellow solid (55.5 mg, 97%). R_f = 2.8/4.0 (20% EtOAc in hexane); ^{1}H NMR (400 MHz, $CDCl_3$) δ 7.43-7.38 (m, 4H), 7.30-7.25 (m, 4H); ^{13}C{^{1}H} NMR ($CDCl_3$, 101 MHz) δ 135.3, 133.8, 129.5, 129.4.

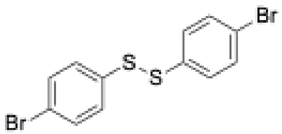

3h

1,2-Bis(4-bromophenyl)disulfane (3h).[2] Following the above procedure with **1h** (102.2 mg, 0.4000 mmol). The crude product was purified by flash column chromatography to afford **3h** as a pale yellow solid (54.2 mg, 72%). R_f = 3.1/4.0 (20% EtOAc in hexane); ^{1}H NMR

(400 MHz, $CDCl_3$) δ 7.45-7.40 (m, 4H), 7.36-7.31 (m, 4H); $^{13}C\{^1H\}$ NMR ($CDCl_3$, 101 MHz) δ 135.9, 132.4, 129.6, 121.7.

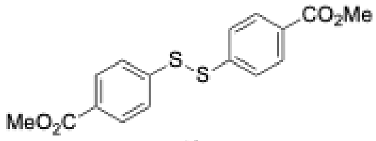

3i

Dimethyl 4,4'-disulfanediyldibenzoate (3i).[5] Following the above procedure with **1i** (93.9 mg, 0.400 mmol). The crude product was purified by flash column chromatography to afford **3i** as a white solid (52.2 mg, 78%). R_f = 1.4/4.0 (20% EtOAc in hexane); ^1H NMR (400 MHz, $CDCl_3$) δ 7.95 (dt, J = 8.8, 2.0 Hz, 4H), 7.51 (dt, J = 8.8, 2.0 Hz, 4H), 3.89 (s, 6H); $^{13}C\{^1H\}$ NMR ($CDCl_3$, 101 MHz) δ 166.5, 142.2, 130.4, 129.0, 126.2, 52.3.

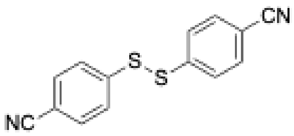

3k

4,4'-Disulfanediyldibenzonitrile (3k).[2] Following the above procedure with **1k** (80.7 mg, 0.400 mmol). The crude product was purified by flash column chromatography to afford **3k** as a pale yellow solid (43.3 mg, 81%). R_f = 1.5/4.0 (20% EtOAc in hexane); ^1H NMR (400 MHz, $CDCl_3$) δ 7.59 (d, J = 8.5 Hz, 4H), 7.55 (d, J = 8.5 Hz, 4H); $^{13}C\{^1H\}$ NMR ($CDCl_3$, 101 MHz) δ 142.2, 132.9, 126.6, 118.2, 111.0.

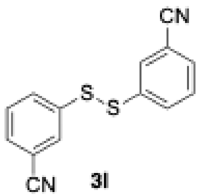

3l

3,3'-Disulfanediyldibenzonitrile (3l).[6] Following the above procedure with **1l** (80.7 mg, 0.400 mmol). The crude product was purified by flash column chromatography to afford **3l** as a pale yellow solid (40.5 mg, 75%). R_f = 1.6/4.0 (20% EtOAc in hexane); ^1H NMR (400 MHz, $CDCl_3$) δ 7.76-7.73 (m, 2H), 7.70-7.66 (m, 2H), 7.56-7.52 (m, 2H), 7.44 (t, J = 7.9 Hz, 2H); $^{13}C\{^1H\}$ NMR ($CDCl_3$, 101 MHz) δ 138.0, 131.3, 131.1, 130.2, 130.1, 117.9, 113.9.

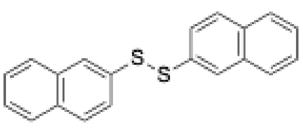

3n

1,2-Di(naphthalen-2-yl)disulfane (3n).[2] Following the above procedure with **1n** (90.7 mg, 0.400 mmol). The crude product was purified by flash column chromatography to afford **3n** as a white solid (52.5 mg, 82%). R_f = 2.6/4.0 (20% EtOAc in hexane); ^1H NMR (400 MHz, $CDCl_3$) δ 8.00 (s, 2H), 7.83-7.77 (m, 4H), 7.76-7.71 (m, 2H), 7.64 (dd, J = 8.6, 1.6 Hz, 2H), 7.50-7.42(m, 4H); $^{13}C\{^1H\}$ NMR ($CDCl_3$, 101 MHz) δ 134.4, 133.6, 132.7, 129.1, 127.9, 127.6, 126.9, 126.7, 126.4, 125.8.

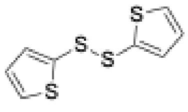

3o

1,2-Di(thiophen-2-yl)disulfane (3o).[4] Following the above procedure with **1o** (73.1 mg, 0.400 mmol). The crude product was purified by flash column chromatography to afford **3o** as a yellow oil (19.9 mg, 42.4%). R_f = 2.4/4.0 (20% EtOAc in hexane); ^1H NMR (400 MHz, $CDCl_3$) δ 7.49 (dd, J = 5.3, 1.2 Hz, 2H), 7.16 (dd, J = 3.6, 1.2 Hz, 2H), 7.02 (dd, J = 5.3, 3.6 Hz, 2H); $^{13}C\{^1H\}$ NMR ($CDCl_3$, 101 MHz) δ 135.9, 135.7, 132.4, 127.9.

Part II: Radical Trapping Experiments with **1a**

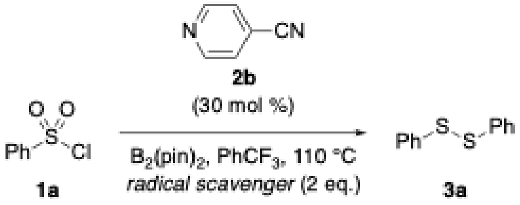

entry	*radical scavenger*	yield of **3a** (%)
1	TEMPO	35
2	BHT	62
3	DPE	N.D.

In a glovebox, an oven-dried vial equipped with a stir bar was charged with **1a** (70.6 mg, 0.400 mmol), radical scavenger (0.800 mmol), $B_2(pin)_2$ (304.7 mg, 1.200 mmol), 4-cyanopyridine (**2b**) (12.5 mg, 0.120 mmol), and dried, degassed benzotrifluoride (2 mL). The vial was sealed with a Telfon-lined cap and stirred in an oil bath preheated to 110 °C for 15 h. After cooling to room temperature, the solvent was removed under reduced pressure and 1,1,2,2-tetrachloroethane (21 μL, 0.20 mmol) was added. The crude mixture was diluted with $CDCl_3$ and analyzed by ^{1}H NMR to determine the yield of **3a**. The ^{1}H NMR sample was then subjected to high-resolution mass spectrometry (HRMS) analysis.

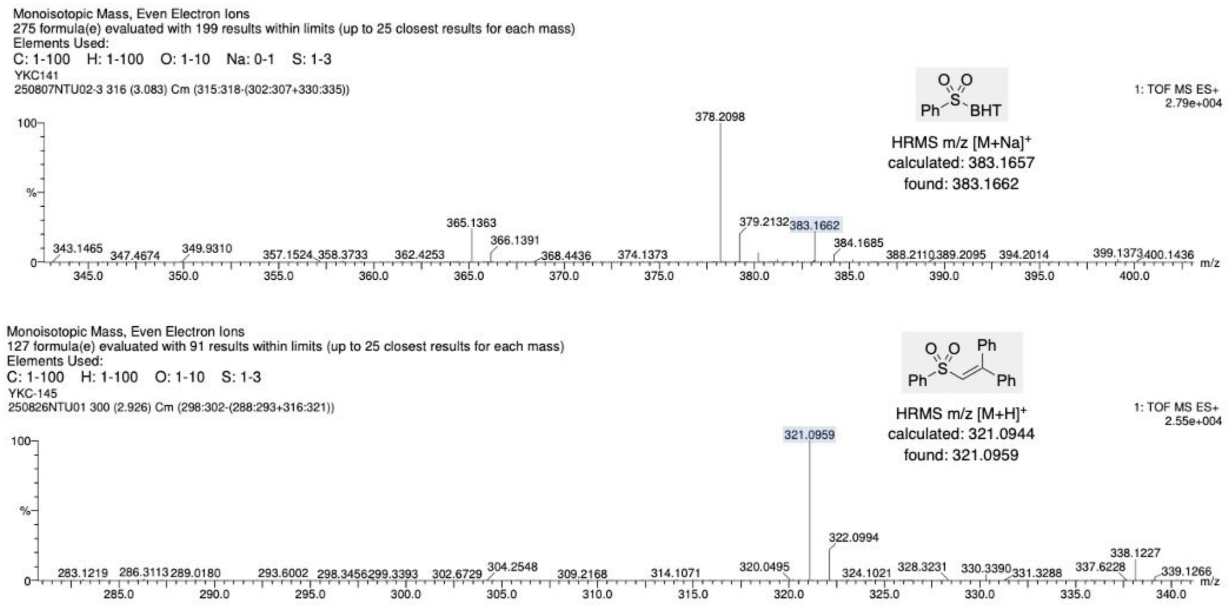

Figure S1. Results of the HRMS analysis from radical trapping experiments using **1a** with butylated hydroxytoluene (BHT) and 1,1-diphenylethylene (DPE)

Part III: Examination of Potential Intermediates **4**–**6** under Optimized Conditions

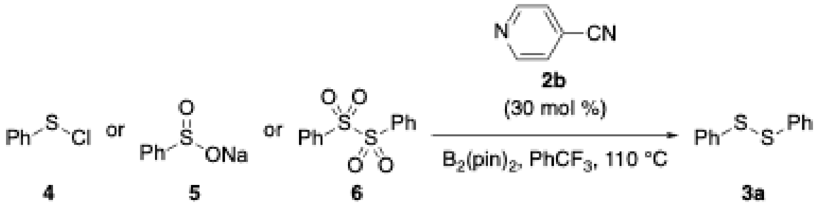

entry	substrate	yield of **3a** (%)
1	**4**	72
2	**5**	N.D.
3	**6**	70

In a glovebox, an oven-dried vial equipped with a stir bar was charged with **4** or **5** or **6** (0.400 mmol), $B_2(pin)_2$ (304.7 mg, 1.200 mmol), 4-cyanopyridine (**2b**) (12.5 mg, 0.120 mmol), and dried, degassed benzotrifluoride (2 mL). The vial was sealed with a Telfon-lined cap and stirred in an oil bath preheated to 110 °C for 15 h. After cooling to room temperature, the solvent was removed under reduced pressure and 1,1,2,2-tetrachloroethane (21 μL, 0.20 mmol) was added. The crude mixture was diluted with $CDCl_3$ and analyzed by ^{1}H NMR to determine the yield of **3a**.

Part IV: Additional Control Experiments

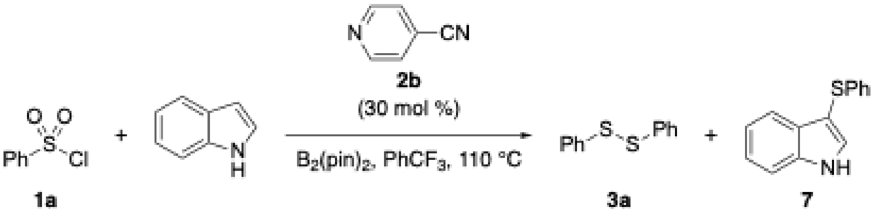

In a glovebox, an oven-dried vial equipped with a stir bar was charged **1a** (70.6 mg, 0.400 mmol), indole (23.4 mg, 0.200 mmol), $B_2(pin)_2$ (304.7 mg, 1.200 mmol), 4-cyanopyridine (**2b**) (12.5 mg, 0.120 mmol), and dried, degassed benzotrifluoride (2 mL). The vial was sealed with a Telfon-lined cap and stirred in an oil bath preheated to 110 °C for 15 h. After cooling to room temperature, the reaction mixture was treated with 1 M NaOH, extracted with DCM, and the combined organic layers were dried over $MgSO_4$ and concentrated under reduced pressure. The crude mixture was purified by flash column chromatography to afford **3a** (17.5 mg, 40%) and **7** (25.1 mg, 56%) as white solids

3-(Phenylthio)-1H-indole (7).[7] R_f = 0.8/4.0 (10% EtOAc in hexane); ^{1}H NMR (400 MHz, $CDCl_3$) δ 8.56 (s, br, 1H), 7.63 (d, *J* = 7.9 Hz, 1H), 7.48 (d, *J* = 2.6 Hz, 1H), 7.27 (td, *J* = 7.1, 1.1 Hz, 1H), δ 7.19-7.10 (m, 5H), 7.05 (tt, *J* = 7.0, 1.5 Hz, 1H); ^{13}C{^{1}H} NMR ($CDCl_3$, 101 MHz) δ 139.4, 136.6, 130.9, 129.2, 128.8, 126.0, 124.9, 123.1, 120.9, 119.7, 111.7, 102.8.

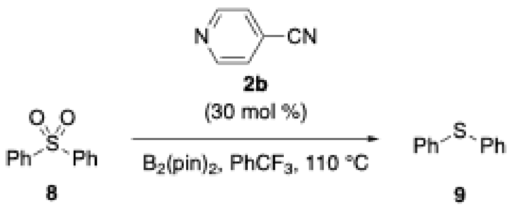

In a glovebox, an oven-dried vial equipped with a stir bar was charged **8** (87.3 mg, 0.400 mmol), $B_2(pin)_2$ (304.7 mg, 1.200 mmol), 4-cyanopyridine (**2b**) (12.5 mg, 0.120 mmol), and dried, degassed benzotrifluoride (2 mL). The vial was sealed with a Telfon-lined cap and stirred in an oil bath preheated to 110 °C for 15 h. After cooling to room temperature, the solvent was removed under reduced pressure and 1,1,2,2-tetrachloroethane (21 μL, 0.20 mmol) was added. The crude mixture was diluted with $CDCl_3$ and analyzed by ^{1}H NMR to determine the yield of **9** (it was not detected).

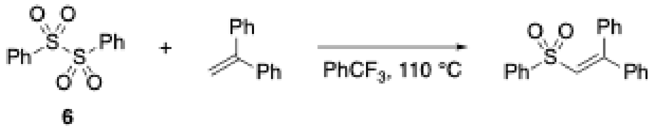

In a glovebox, an oven-dried vial equipped with a stir bar was charged with **6** (56.5 mg, 0.200 mmol), 1,1-diphenylethylene (72.1 mg, 0.400 mmol) and dried, degassed benzotrifluoride (1 mL). The vial was sealed with a Telfon-lined cap and stirred in an oil bath preheated to 110 °C for 15 h. After cooling to room temperature, the solvent was removed under reduced pressure. The crude mixture was diluted with $CDCl_3$ and analyzed by ^{1}H NMR. The ^{1}H NMR sample was then subjected to high-resolution mass spectrometry (HRMS) analysis.

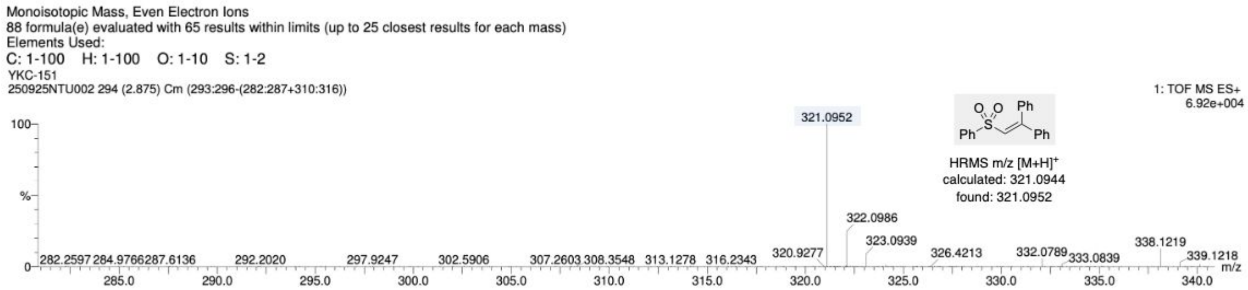

Figure S2. Result of the HRMS analysis from radical trapping experiment using **6** with 1,1-diphenylethylene (DPE)

Part V: References

1. Rodrigo, S.; Hazra, A.; Mahajan, J. P.; Nguyen, H. M.; Luo, L. *J. Am. Chem. Soc.* **2023**, *145*, 21851–21859.
2. Zhou, W.; Le, L.; Chen, Y.; Xie, W.; Chen, Y.; Yin, S.-F.; Qiu, R. *J. Org. Chem.* **2025**, *90*, 2927–2936.

3. Weierbach, S. M.; Reynolds, R. P.; Stephens, S. M.; Vlasakakis, K V.; Ritter, R. T.; White, O. M.; Patel, N. H.; Hayes, E. C.; Dunmire, S.; Lambert, K. M. *J. Org. Chem.* **2023**, *88*, 11392–11410.
4. Wang, L.; Qin, Z.; Chen. L.; Qin, X.; Hou, J.; Wang, C.; Li, X.; Duan, H.; Fang, B.; Wang, M.; An, J. *Molecules* **2024**, *29*, 3361.
5. Zheng, Y.; Qing, F.-L.; Huang, Y.; Xu, H.-H. *Adv. Synth. Catal.* **2016**, *358*, 3477–3481.
6. Ajenjo, J.; Klepetářová, B.; Greenhall, M.; Bím, D.; Culka, M.; Rulíšek, L.; Beier, P. *Eur. J. Chem.* **2019**, *25*, 11375–11382.
7. Ghosh, A.; Lecomte, M.; Kim-Lee, S.-H.; Radosevich, A. T. *Angew. Chem. Int. Ed.* **2019**, *58*, 2864–2869.

Part VI: Copies of NMR Spectra Data

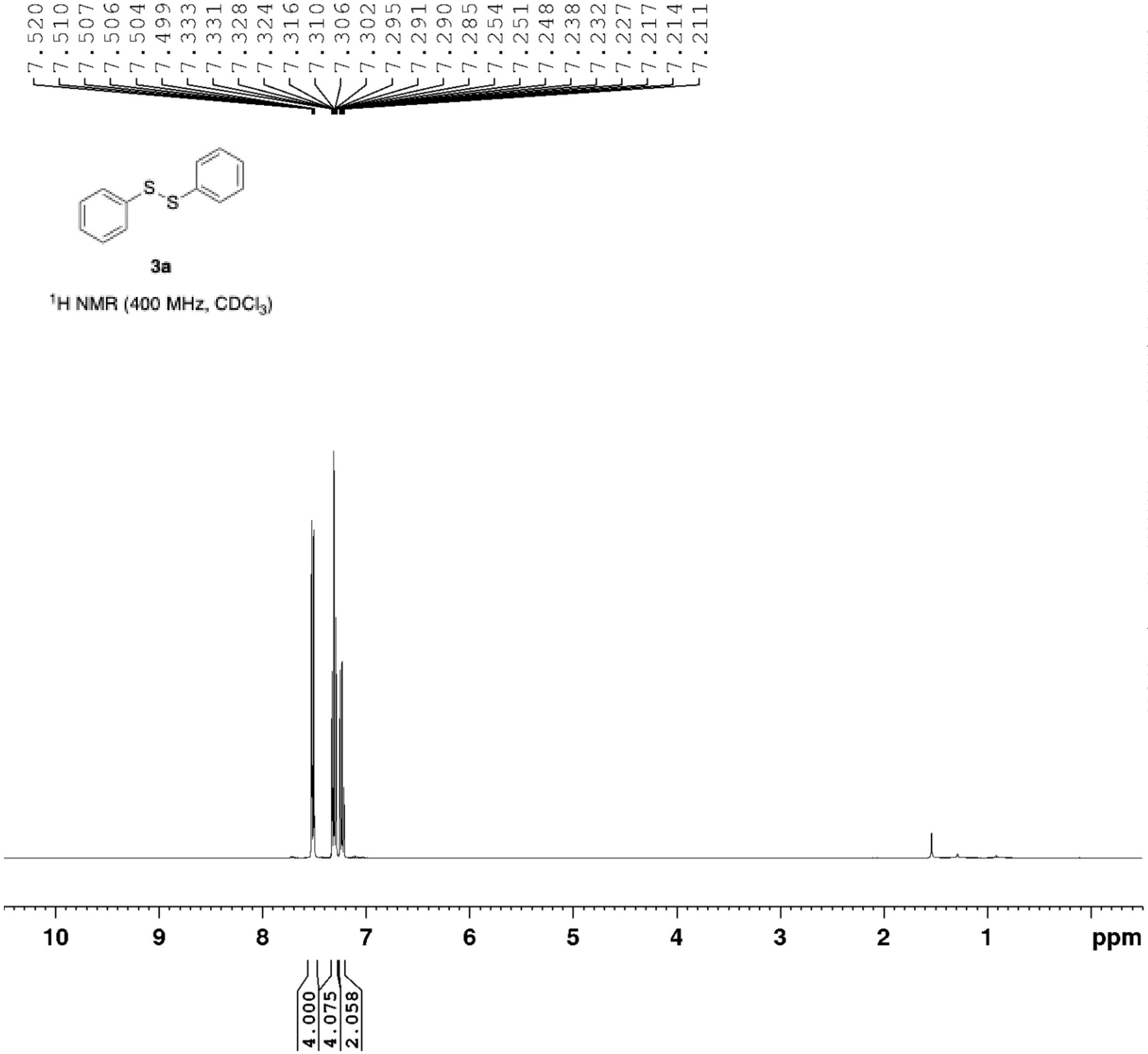


```
Current Data Parameters
NAME                    YKC-172-3a
EXPNO                            1
PROCNO                           1

F2 - Acquisition Parameters
Date_                     20251128
Time                         12.25 h
INSTRUM                      spect
PROBHD      Z108618_0411
PULPROG                       zg30
TD                           32768
SOLVENT                      CDCl3
NS                              16
DS                               0
SWH                       8802.817 Hz
FIDRES                    0.537281 Hz
AQ                       1.8612224 sec
RG                          103.36
DW                          56.800 usec
DE                           15.08 usec
TE                           298.0 K
D1                      1.00000000 sec
TD0                              1
SFO1                   400.1328009 MHz
NUC1                            1H
P0                            5.17 usec
P1                           15.50 usec
PLW1                    9.89999962 W

F2 - Processing parameters
SI                          131072
SF                     400.1300135 MHz
WDW                             EM
SSB                              0
LB                               0 Hz
GB                               0
PC                            1.00
```

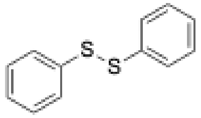

3a

$^{13}C\{^{1}H\}$ NMR (400 MHz, $CDCl_3$)

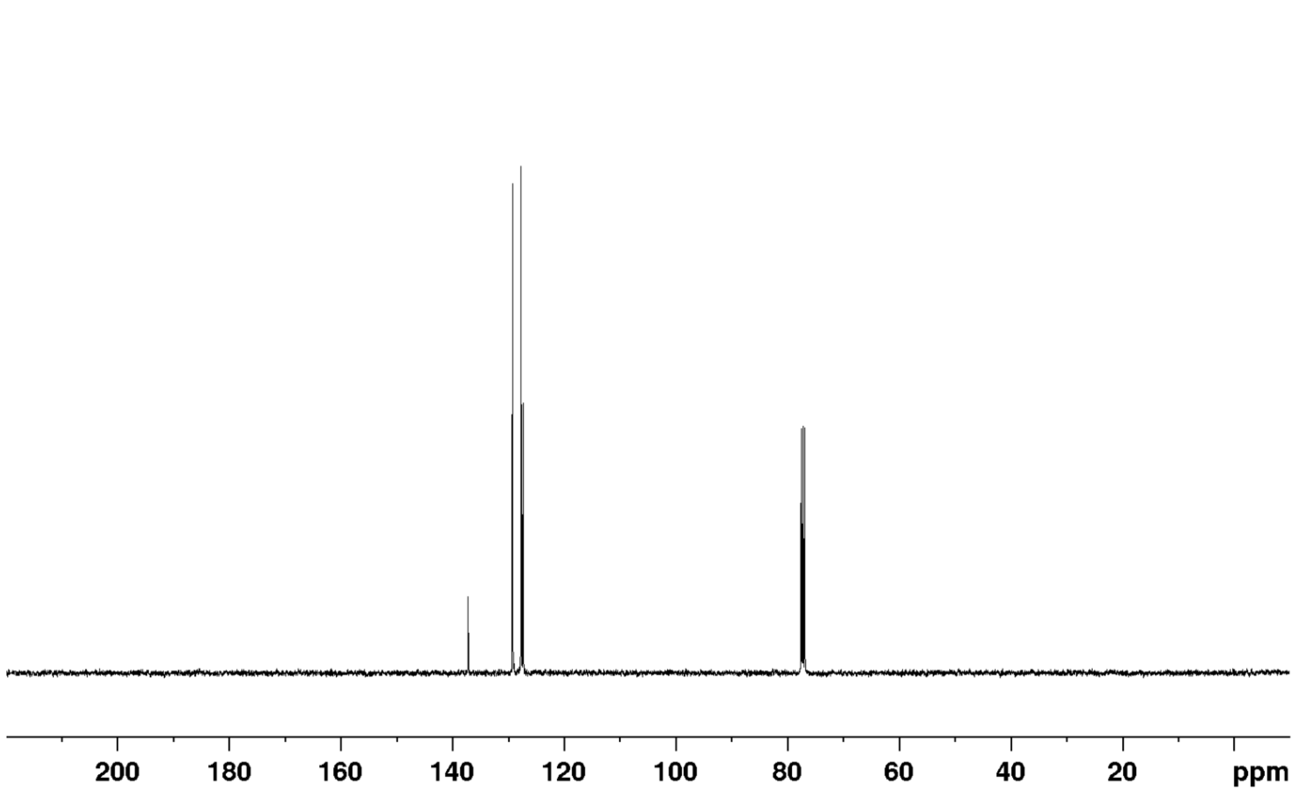


```
Current Data Parameters
NAME                 YKC-172-3a
EXPNO                         2
PROCNO                        1

F2 - Acquisition Parameters
Date_                  20251128
Time                      12.32 h
INSTRUM                   spect
PROBHD       Z108618_0411
PULPROG                 zgpg30
TD                        65536
SOLVENT                   CDCl3
NS                          100
DS                            0
SWH                   28409.092 Hz
FIDRES                 0.866977 Hz
AQ                    1.1534336 sec
RG                       212.49
DW                       17.600 usec
DE                         6.50 usec
TE                        298.1 K
D1                   2.00000000 sec
D11                  0.03000000 sec
TD0                           1
SFO1               100.6258487 MHz
NUC1                        13C
P0                         3.33 usec
P1                        10.00 usec
PLW1                37.00000000 W
SFO2               400.1316005 MHz
NUC2                         1H
CPDPRG[2]               waltz16
PCPD2                     90.00 usec
PLW2                 9.89999962 W
PLW12                0.29363999 W
PLW13                0.14770000 W

F2 - Processing parameters
SI                        32768
SF                 100.6127596 MHz
WDW                          EM
SSB                           0
LB                         3.00 Hz
GB                            0
PC                         1.40
```

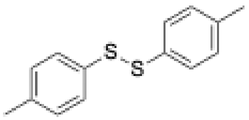

3b

^{1}H NMR (400 MHz, $CDCl_3$)

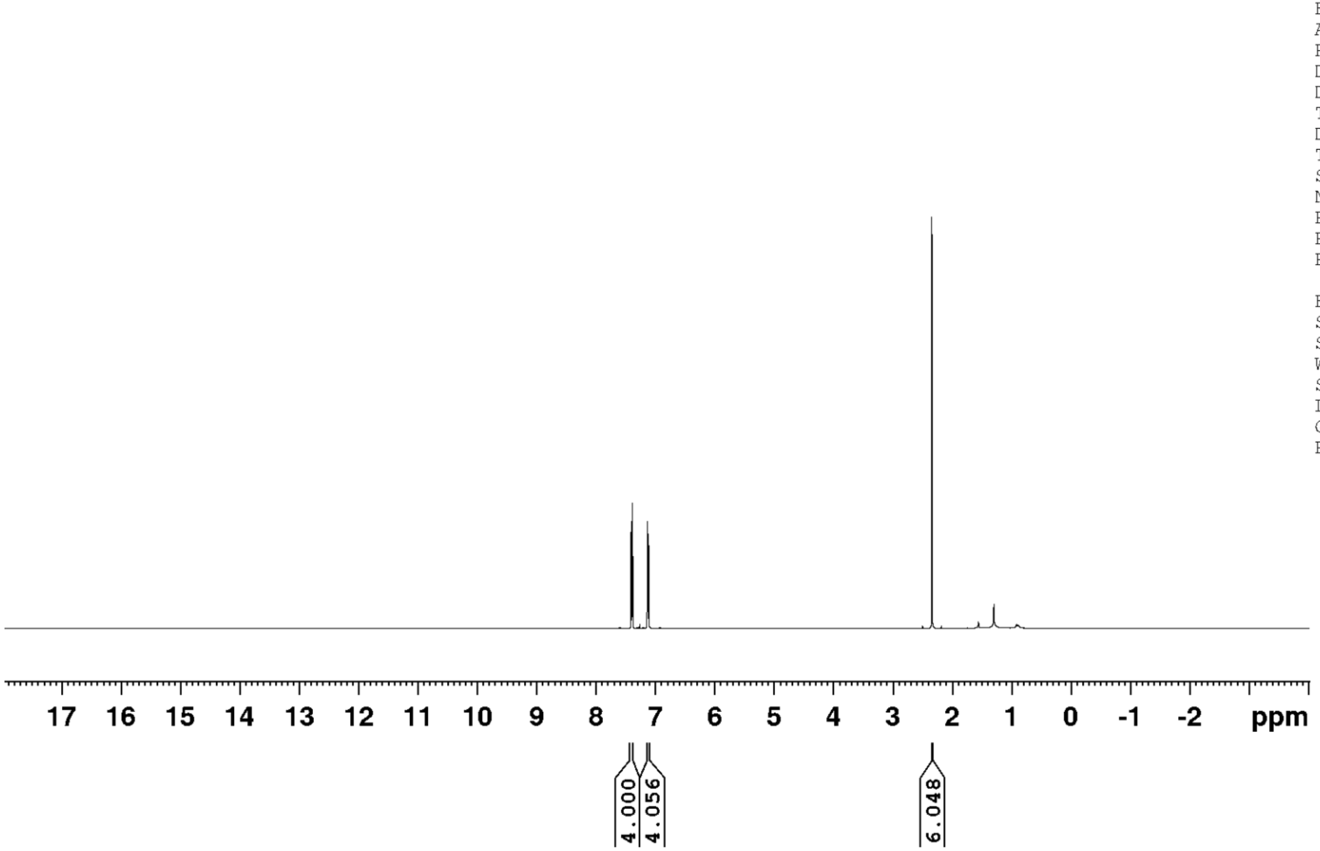

Current Data Parameters		
NAME	YKC-127-3b	
EXPNO	1	
PROCNO	1	
F2 - Acquisition Parameters		
Date_	20250703	
Time	17.44	h
INSTRUM	spect	
PROBHD	Z108618_0411	
PULPROG	zg30	
TD	32768	
SOLVENT	CDCl3	
NS	16	
DS	0	
SWH	8802.817	Hz
FIDRES	0.537281	Hz
AQ	1.8612224	sec
RG	71.05	
DW	56.800	usec
DE	15.08	usec
TE	298.0	K
D1	1.00000000	sec
TD0	1	
SFO1	400.1328009	MHz
NUC1	1H	
P0	5.17	usec
P1	15.50	usec
PLW1	9.89999962	W
F2 - Processing parameters		
SI	131072	
SF	400.1300096	MHz
WDW	EM	
SSB	0	
LB	0	Hz
GB	0	
PC	1.00	

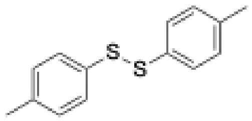

3b

$^{13}C\{^{1}H\}$ NMR (400 MHz, $CDCl_3$)

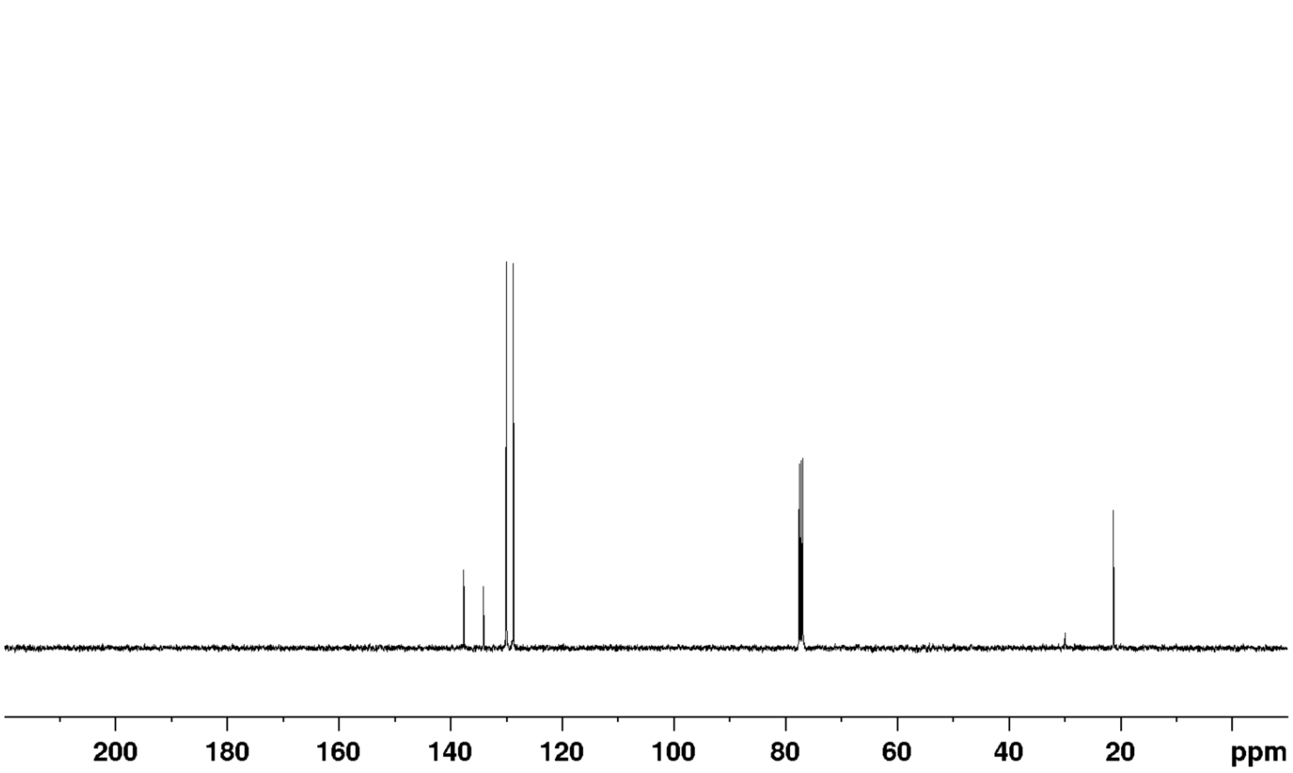

Current Data Parameters

NAME	YKC-127-3b
EXPNO	2
PROCNO	1

F2 - Acquisition Parameters

Date_	20250703
Time	17.49 h
INSTRUM	spect
PROBHD	Z108618_0411
PULPROG	zgpg30
TD	65536
SOLVENT	CDCl3
NS	65
DS	0
SWH	28409.092 Hz
FIDRES	0.866977 Hz
AQ	1.1534336 sec
RG	212.49
DW	17.600 usec
DE	6.50 usec
TE	298.1 K
D1	2.00000000 sec
D11	0.03000000 sec
TD0	1
SFO1	100.6258487 MHz
NUC1	13C
P0	3.50 usec
P1	10.50 usec
PLW1	42.50000000 W
SFO2	400.1316005 MHz
NUC2	1H
CPDPRG[2]	waltz16
PCPD2	90.00 usec
PLW2	9.89999962 W
PLW12	0.29363999 W
PLW13	0.14770000 W

F2 - Processing parameters

SI	32768
SF	100.6127592 MHz
WDW	EM
SSB	0
LB	3.00 Hz
GB	0
PC	1.40

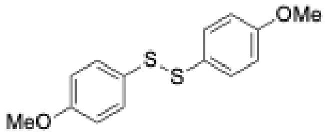

3c

^{1}H NMR (400 MHz, $CDCl_3$)

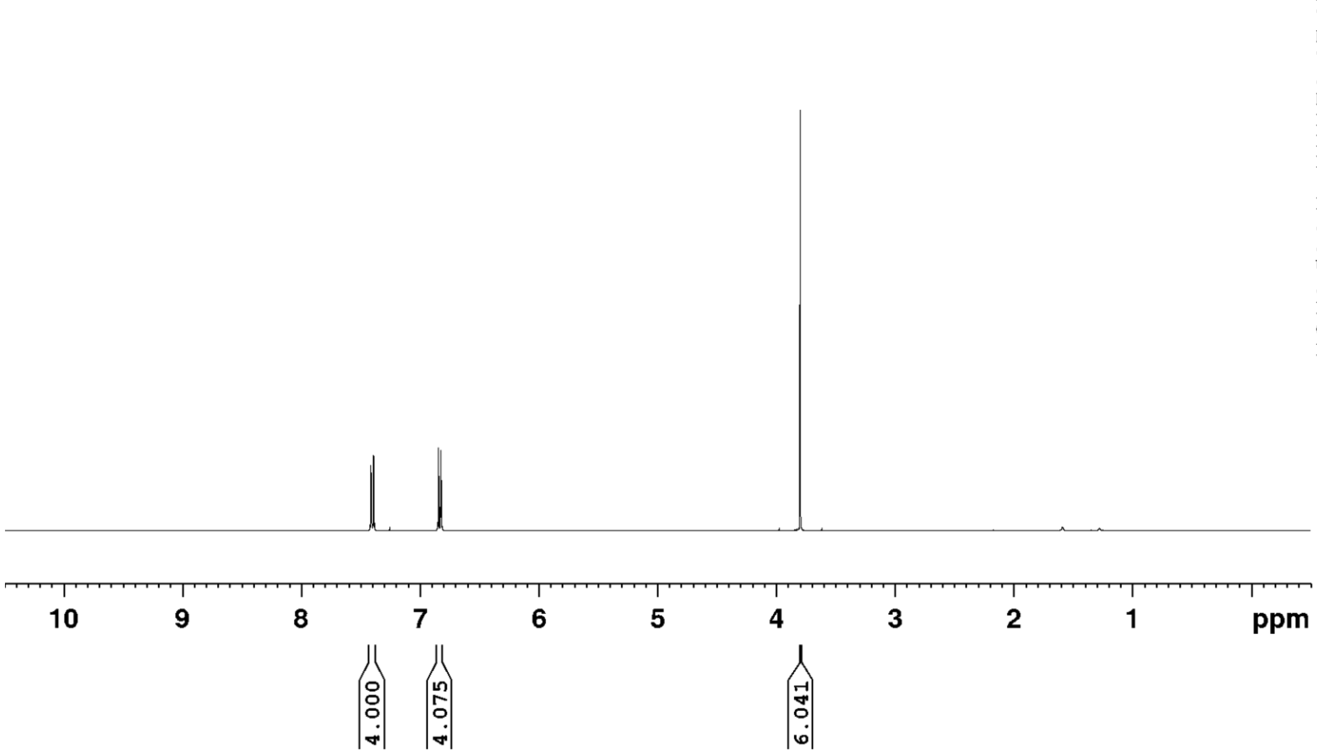

Current Data Parameters

NAME	YKC-121-3c
EXPNO	1
PROCNO	1

F2 - Acquisition Parameters

Date_	20250623
Time	14.30 h
INSTRUM	spect
PROBHD	Z108618_0411
PULPROG	zg30
TD	32768
SOLVENT	CDCl3
NS	16
DS	0
SWH	8802.817 Hz
FIDRES	0.537281 Hz
AQ	1.8612224 sec
RG	103.36
DW	56.800 usec
DE	15.08 usec
TE	301.5 K
D1	1.00000000 sec
TD0	1
SFO1	400.1328009 MHz
NUC1	1H
P0	5.17 usec
P1	15.50 usec
PLW1	9.89999962 W

F2 - Processing parameters

SI	131072
SF	400.1300095 MHz
WDW	EM
SSB	0
LB	0 Hz
GB	0
PC	1.00

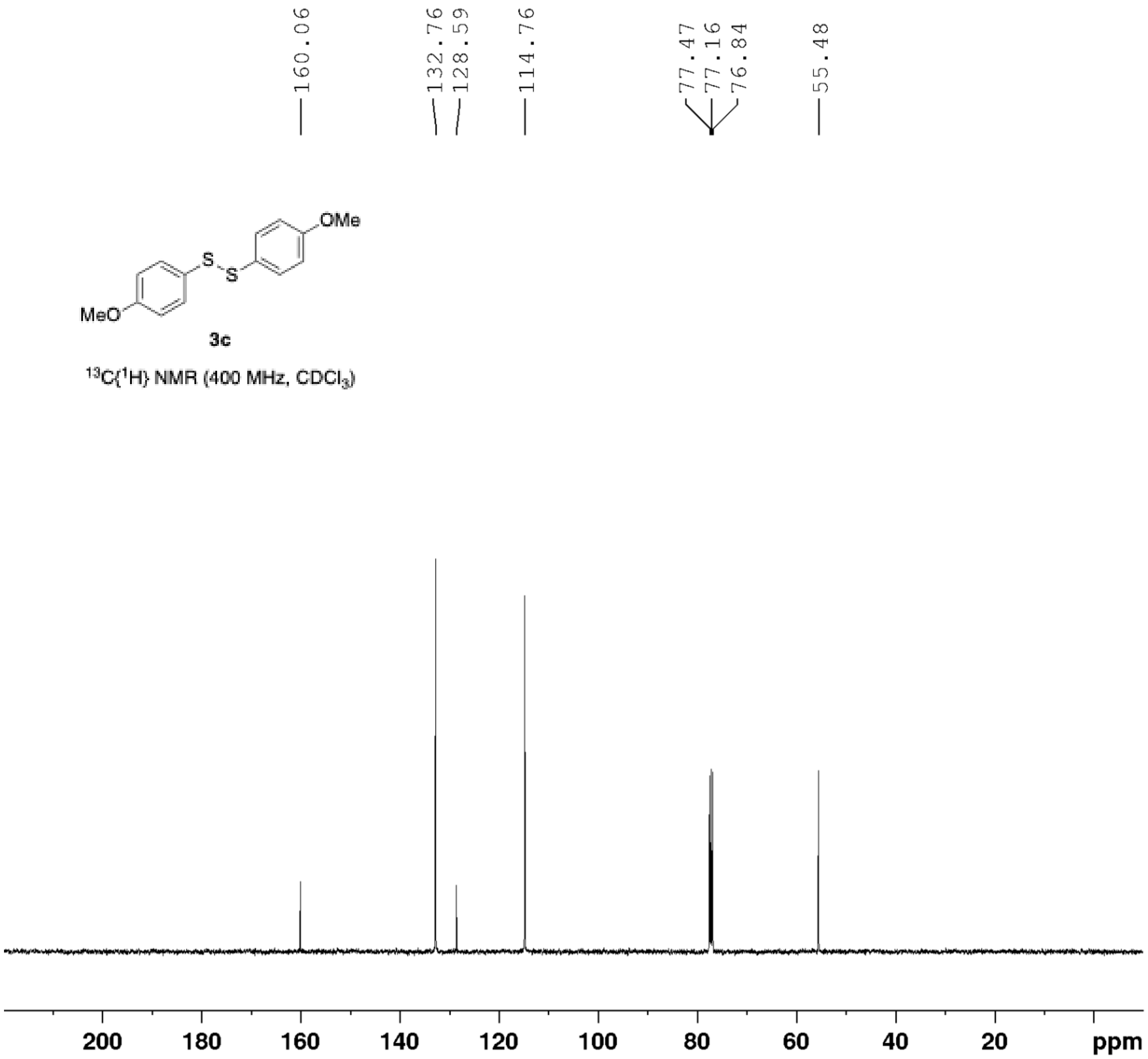


```
Current Data Parameters
NAME             YKC-121-3c
EXPNO                     2
PROCNO                    1

F2 - Acquisition Parameters
Date_              20250623
Time                  14.36 h
INSTRUM               spect
PROBHD      Z108618_0411
PULPROG              zgpg30
TD                    65536
SOLVENT               CDCl3
NS                       94
DS                        0
SWH               28409.092 Hz
FIDRES             0.866977 Hz
AQ                1.1534336 sec
RG                   212.49
DW                   17.600 usec
DE                     6.50 usec
TE                    301.6 K
D1               2.00000000 sec
D11              0.03000000 sec
TD0                       1
SFO1            100.6258487 MHz
NUC1                    13C
P0                     3.50 usec
P1                    10.50 usec
PLW1            42.50000000 W
SFO2            400.1316005 MHz
NUC2                     1H
CPDPRG[2]           waltz16
PCPD2                 90.00 usec
PLW2             9.89999962 W
PLW12            0.29363999 W
PLW13            0.14770000 W

F2 - Processing parameters
SI                    32768
SF              100.6127584 MHz
WDW                      EM
SSB                       0
LB                     3.00 Hz
GB                        0
PC                     1.40
```

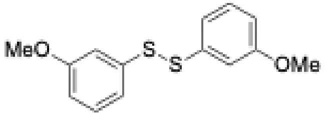

3d

^{1}H NMR (400 MHz, $CDCl_3$)

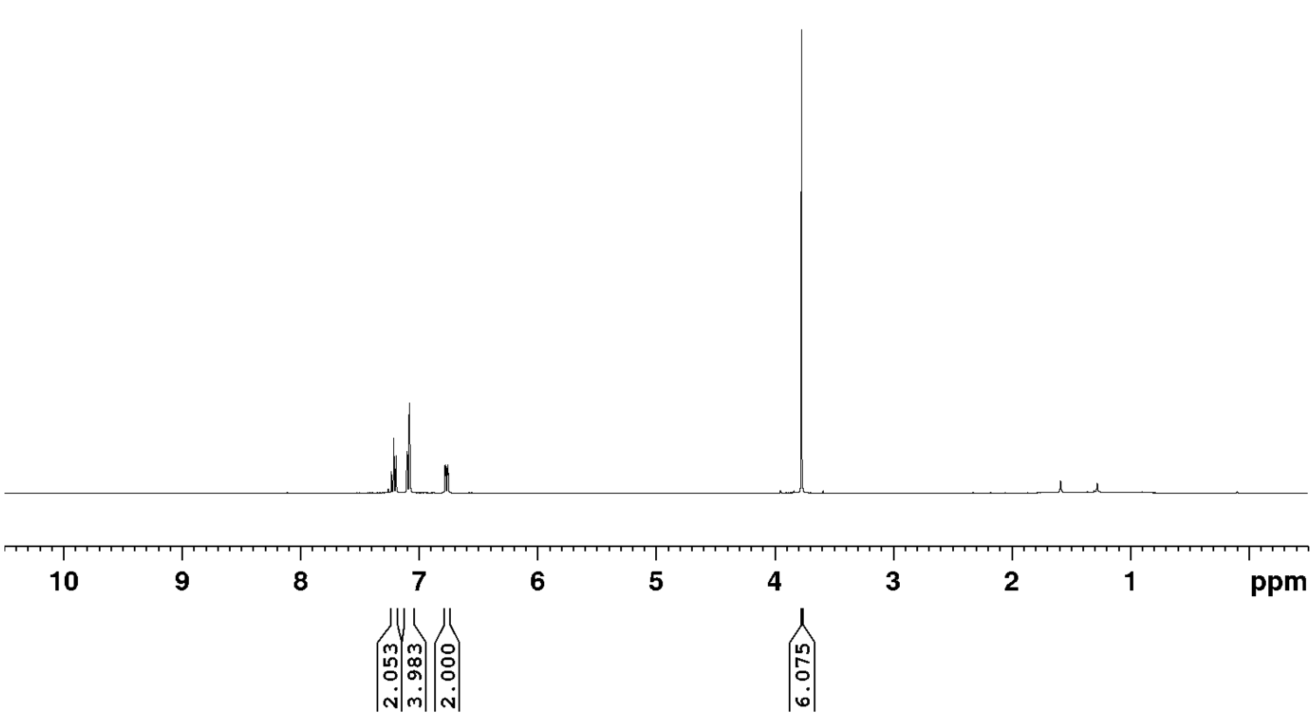


```
Current Data Parameters
NAME              YKC-128-3d
EXPNO                      1
PROCNO                     1

F2 - Acquisition Parameters
Date_               20250709
Time                   17.43 h
INSTRUM                spect
PROBHD      Z108618_0411
PULPROG                 zg30
TD                     32768
SOLVENT                CDCl3
NS                        16
DS                         0
SWH                 8802.817 Hz
FIDRES              0.537281 Hz
AQ                 1.8612224 sec
RG                     94.01
DW                    56.800 usec
DE                     15.08 usec
TE                     298.0 K
D1                1.00000000 sec
TD0                        1
SFO1           400.1328009 MHz
NUC1                      1H
P0                      5.17 usec
P1                     15.50 usec
PLW1              9.89999962 W

F2 - Processing parameters
SI                    131072
SF             400.1300096 MHz
WDW                       EM
SSB                        0
LB                         0 Hz
GB                         0
PC                      1.00
```

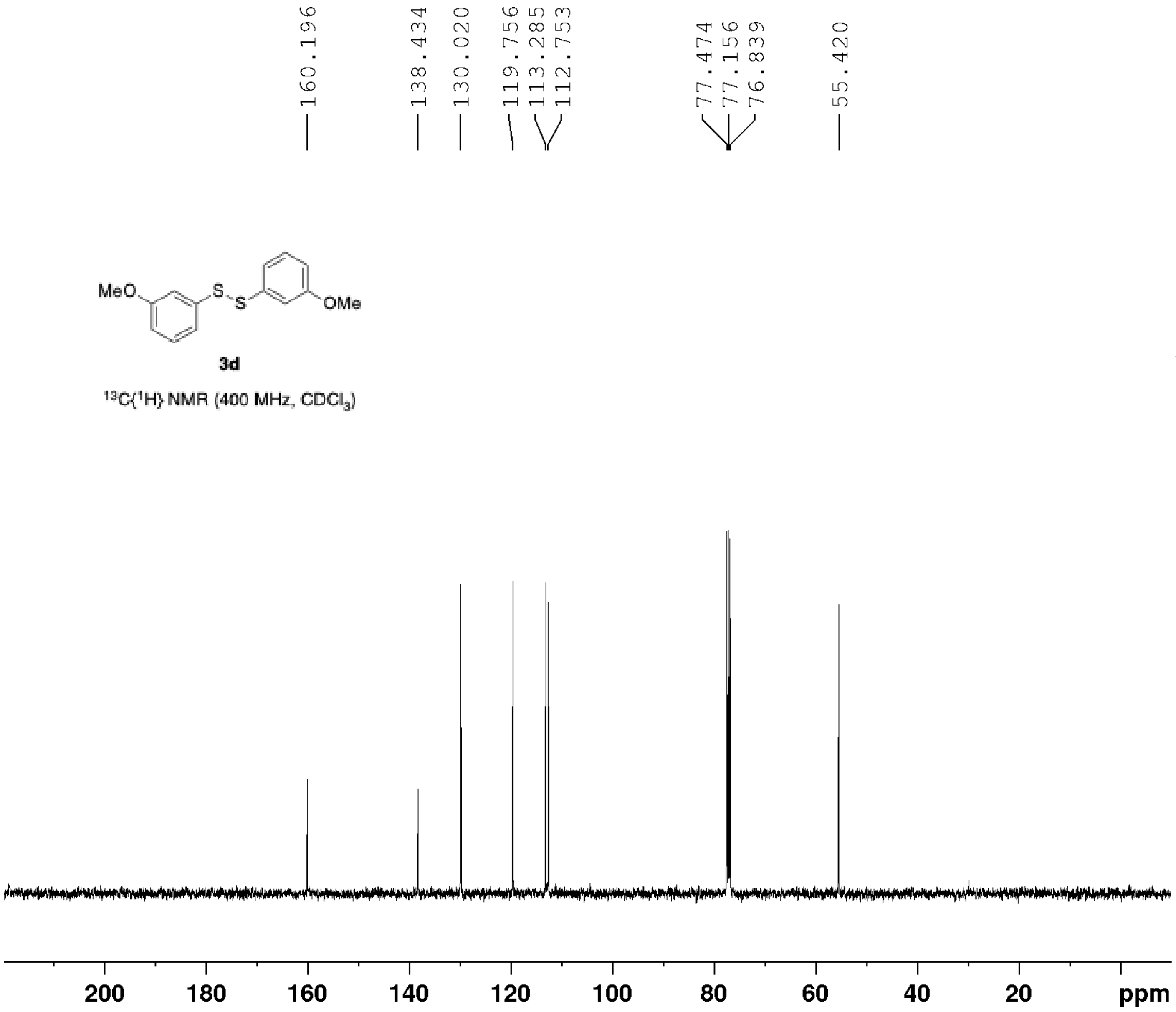

3d

$^{13}C\{^1H\}$ NMR (400 MHz, $CDCl_3$)

Current Data Parameters

NAME	YKC-128-3d	
EXPNO	2	
PROCNO	1	

F2 - Acquisition Parameters

Date_	20250709	
Time	17.47	h
INSTRUM	spect	
PROBHD	Z108618_0411	
PULPROG	zgpg30	
TD	65536	
SOLVENT	CDCl3	
NS	65	
DS	0	
SWH	28409.092	Hz
FIDRES	0.866977	Hz
AQ	1.1534336	sec
RG	212.49	
DW	17.600	usec
DE	6.50	usec
TE	298.1	K
D1	2.00000000	sec
D11	0.03000000	sec
TD0	1	
SFO1	100.6258487	MHz
NUC1	13C	
P0	3.50	usec
P1	10.50	usec
PLW1	42.50000000	W
SFO2	400.1316005	MHz
NUC2	1H	
CPDPRG[2]	waltz16	
PCPD2	90.00	usec
PLW2	9.89999962	W
PLW12	0.29363999	W
PLW13	0.14770000	W

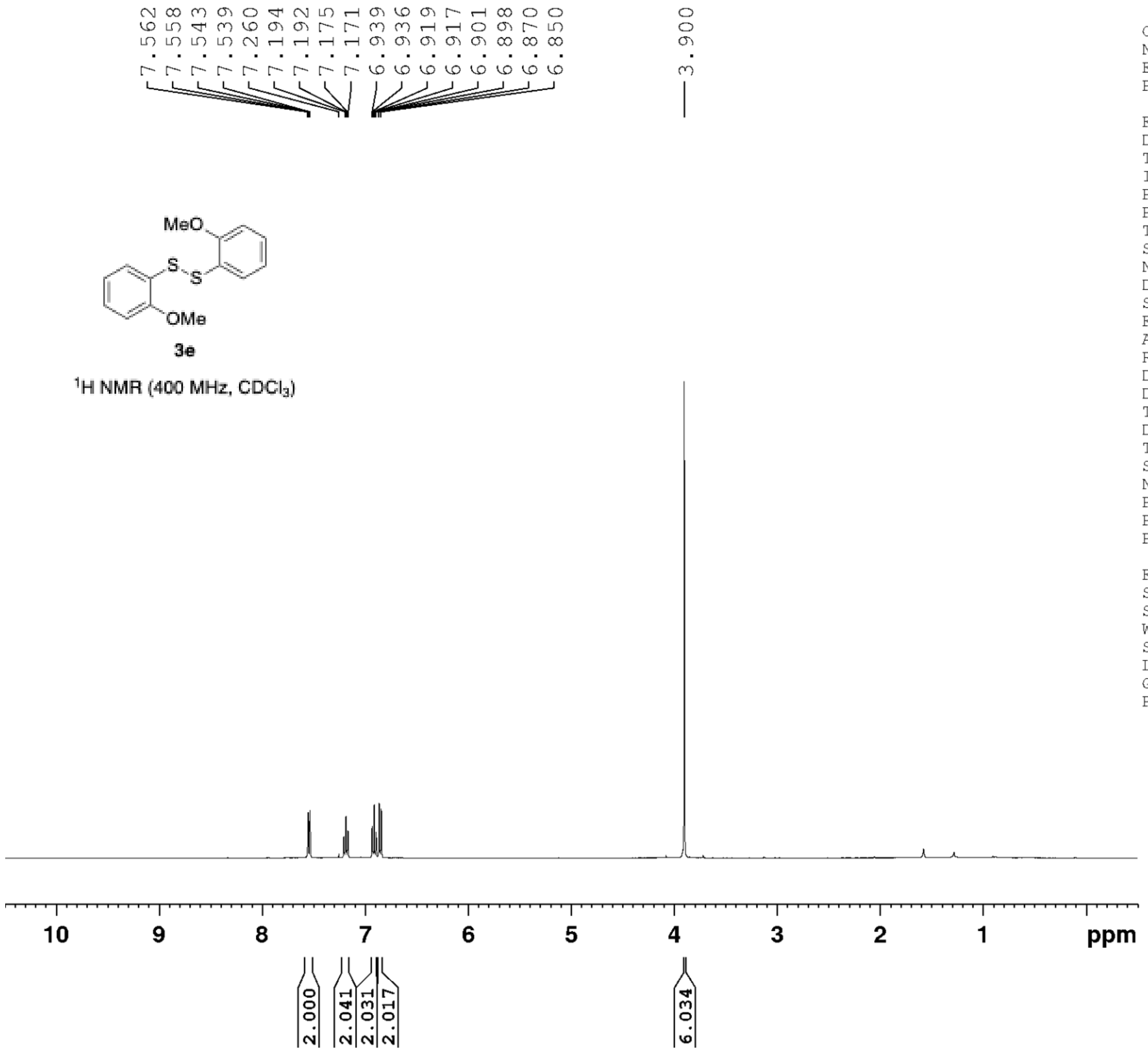

Current Data Parameters
NAME YKC-130-3e
EXPNO 1
PROCNO 1

F2 - Acquisition Parameters
Date_ 20250711
Time 17.48 h
INSTRUM spect
PROBHD Z108618_0411
PULPROG zg30
TD 32768
SOLVENT CDCl3
NS 16
DS 0
SWH 8802.817 Hz
FIDRES 0.537281 Hz
AQ 1.8612224 sec
RG 83.88
DW 56.800 usec
DE 15.08 usec
TE 298.0 K
D1 1.00000000 sec
TD0 1
SFO1 400.1328009 MHz
NUC1 1H
P0 5.17 usec
P1 15.50 usec
PLW1 9.89999962 W

F2 - Processing parameters
SI 131072
SF 400.1300098 MHz
WDW EM
SSB 0
LB 0 Hz
GB 0
PC 1.00

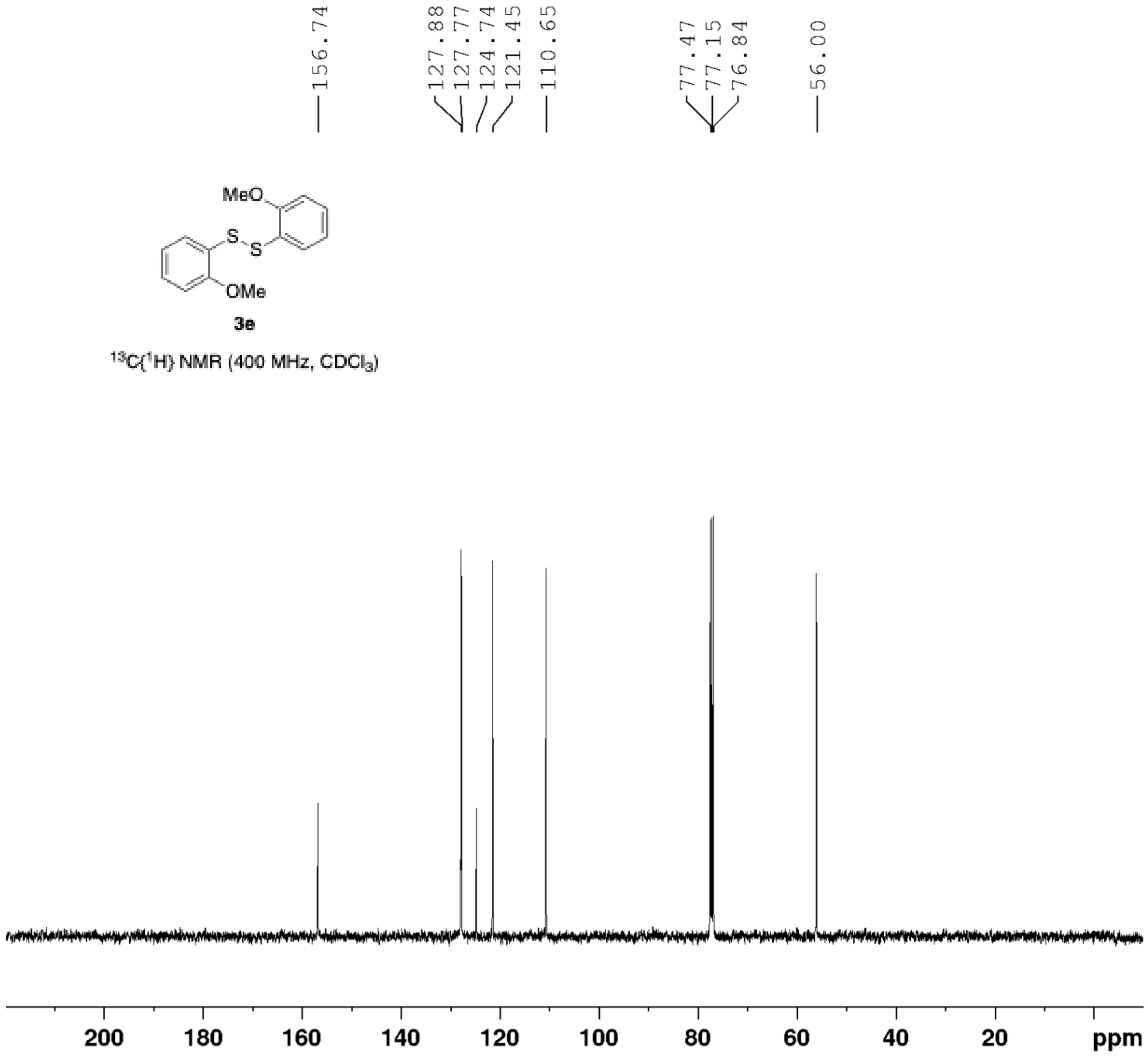

Current Data Parameters
NAME YKC-130-3e
EXPNO 2
PROCNO 1

F2 - Acquisition Parameters
Date_ 20250711
Time 17.52 h
INSTRUM spect
PROBHD Z108618_0411
PULPROG zgpg30
TD 65536
SOLVENT CDCl3
NS 60
DS 0
SWH 28409.092 Hz
FIDRES 0.866977 Hz
AQ 1.1534336 sec
RG 212.49
DW 17.600 usec
DE 6.50 usec
TE 298.0 K
D1 2.00000000 sec
D11 0.03000000 sec
TD0 1
SFO1 100.6258487 MHz
NUC1 13C
P0 3.50 usec
P1 10.50 usec
PLW1 42.50000000 W
SFO2 400.1316005 MHz
NUC2 1H
CPDPRG[2] waltz16
PCPD2 90.00 usec
PLW2 9.89999962 W
PLW12 0.29363999 W
PLW13 0.14770000 W

F2 - Processing parameters
SI 32768
SF 100.6127610 MHz
WDW EM
SSB 0
LB 3.00 Hz
GB 0
PC 1.40

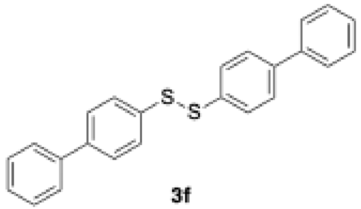

3f

^{1}H NMR (400 MHz, $CDCl_3$)

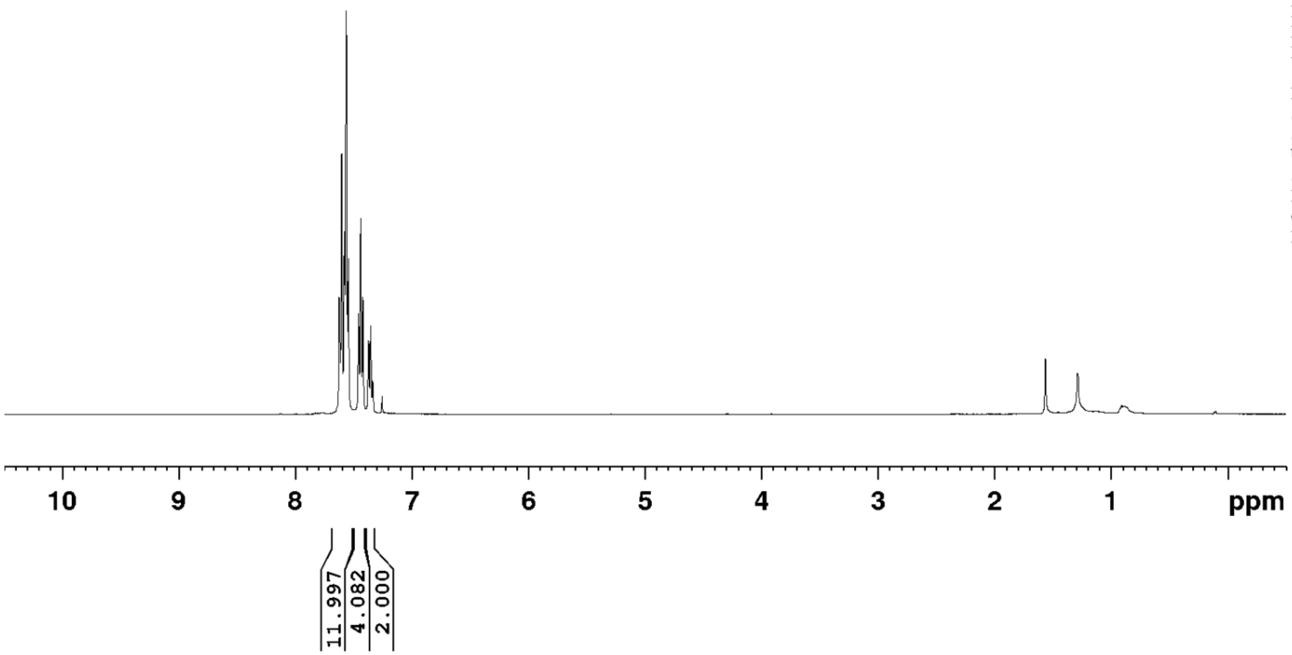


```
Current Data Parameters
NAME                YKC-124-3f
EXPNO                        1
PROCNO                       1

F2 - Acquisition Parameters
Date_                 20250625
Time                     12.57
INSTRUM         Avance NEO 400
PROBHD            Z104450_0301
PULPROG                   zg30
TD                       32768
SOLVENT                  CDCl3
NS                          16
DS                           0
SWH                   8196.721
FIDRES                0.500288
AQ                   1.9988480
RG                         101
DW                       0.000
DE                        6.50
TE                       302.2
D1                  1.00000000
TD0                          1
SFO1               400.1528010
NUC1                        1H
P0                        5.00
P1                       15.00
PLW1                8.71050000

F2 - Processing parameters
SI                       32768
SF                 400.1500095 MHz
WDW                         EM
SSB                          0
LB                        0.30 Hz
GB                           0
PC                        1.00
```

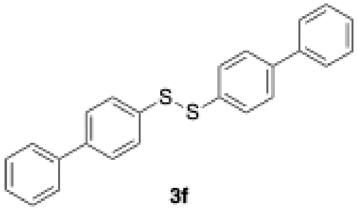

3f

$^{13}C\{^{1}H\}$ NMR (400 MHz, $CDCl_3$)

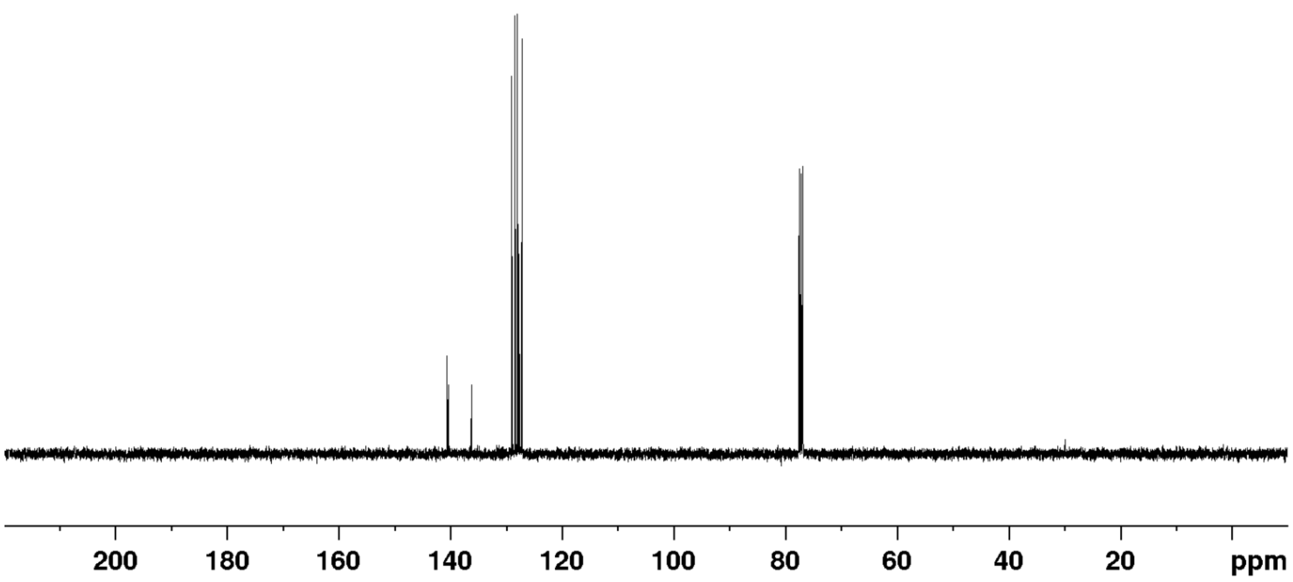

Current Data Parameters
NAME YKC-124-3f
EXPNO 2
PROCNO 1

F2 - Acquisition Parameters
Date_ 20250625
Time 13.05
INSTRUM Avance NEO 400
PROBHD Z104450_0301
PULPROG zgpg30
TD 65536
SOLVENT CDCl3
NS 100
DS 0
SWH 28901.734
FIDRES 0.882011
AQ 1.1337728
RG 14.2
DW 0.000
DE 6.50
TE 302.4
D1 2.00000000
D11 0.03000000
TD0 1
SFO1 100.6298707
NUC1 13C
P0 3.33
P1 10.00
PLW1 48.26600000
SFO2 400.1516006
NUC2 1H
CPDPRG2 waltz16
PCPD2 90.00
PLW2 8.71050000
PLW12 0.24196000
PLW13 0.12170000

F2 - Processing parameters
SI 32768
SF 100.6177859 MHz
WDW EM
SSB 0
LB 1.00 Hz
GB 0
PC 1.40

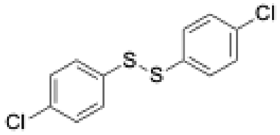

3g

^{1}H NMR (400 MHz, $CDCl_3$)

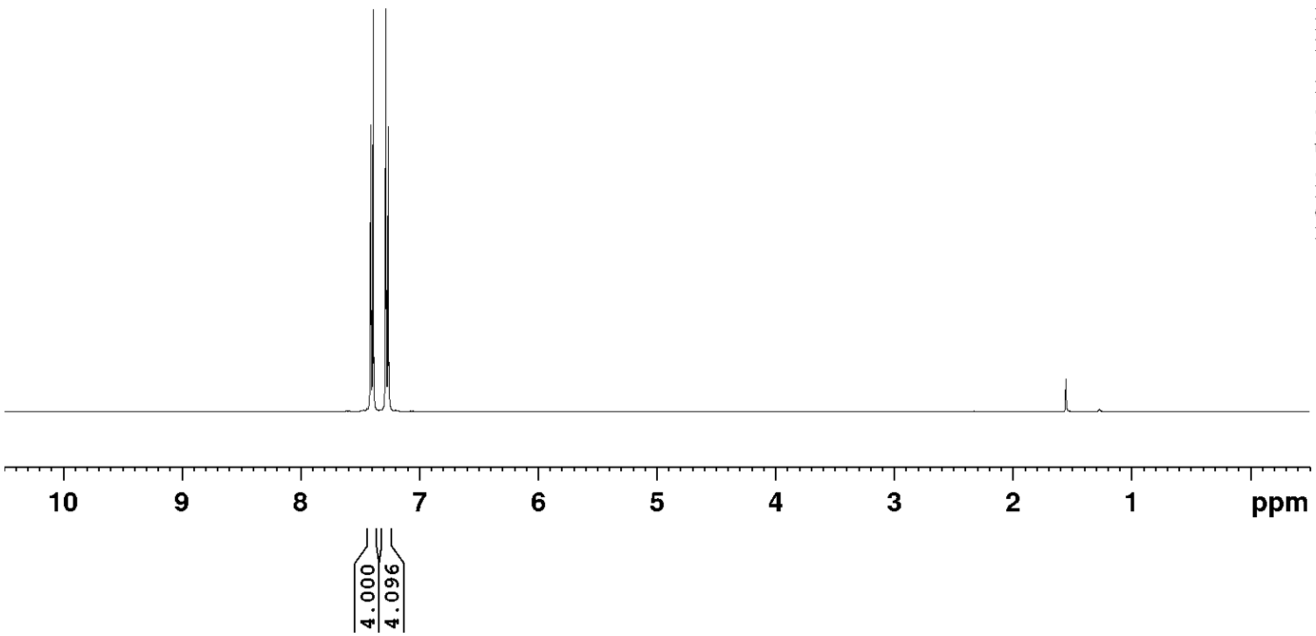


```
Current Data Parameters
NAME                 YKC-175-3g
EXPNO                         1
PROCNO                        1

F2 - Acquisition Parameters
Date_                  20251127
Time                      12.01
INSTRUM          Avance NEO 400
PROBHD             Z104450_0301
PULPROG                    zg30
TD                        32768
SOLVENT                   CDCl3
NS                           16
DS                            0
SWH                    8196.721
FIDRES                 0.500288
AQ                    1.9988480
RG                          101
DW                        0.000
DE                         6.50
TE                        302.4
D1                   1.00000000
TD0                           1
SFO1                400.1528010
NUC1                         1H
P0                         5.00
P1                        15.00
PLW1                 8.71050000

F2 - Processing parameters
SI                        32768
SF                  400.1500095 MHz
WDW                          EM
SSB                           0
LB                         0.30 Hz
GB                            0
PC                         1.00
```

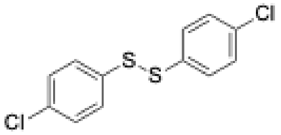

3g

$^{13}C\{^{1}H\}$ NMR (400 MHz, $CDCl_3$)

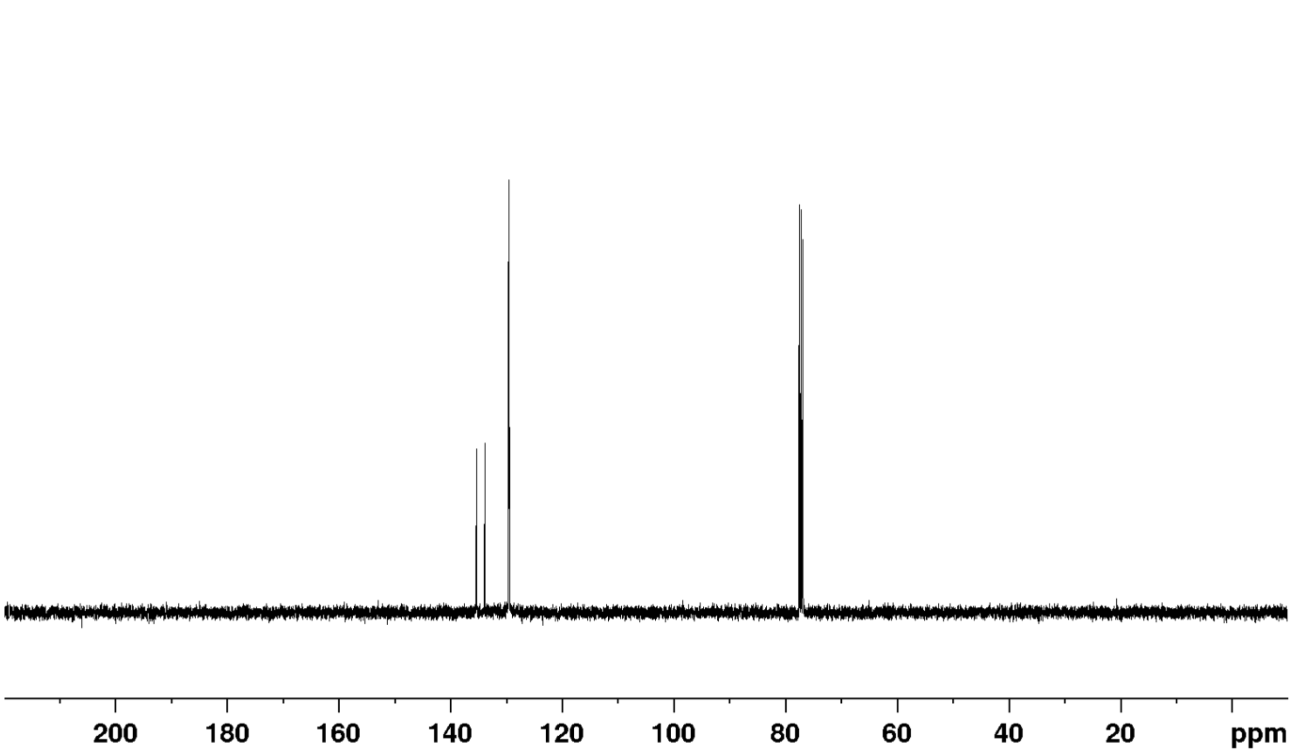

Current Data Parameters

NAME	YKC-175-3g
EXPNO	2
PROCNO	1

F2 - Acquisition Parameters

Date_	20251127
Time	12.07
INSTRUM	Avance NEO 400
PROBHD	Z104450_0301
PULPROG	zgpg30
TD	65536
SOLVENT	CDCl3
NS	100
DS	0
SWH	28901.734
FIDRES	0.882011
AQ	1.1337728
RG	16
DW	0.000
DE	6.50
TE	302.6
D1	2.00000000
D11	0.03000000
TD0	1
SFO1	100.6298707
NUC1	13C
P0	3.33
P1	10.00
PLW1	48.26600000
SFO2	400.1516006
NUC2	1H
CPDPRG2	waltz16
PCPD2	90.00
PLW2	8.71050000
PLW12	0.24196000
PLW13	0.12170000

F2 - Processing parameters

SI	32768
SF	100.6177861 MHz
WDW	EM
SSB	0
LB	1.00 Hz
GB	0
PC	1.40

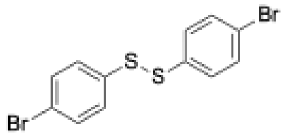

3h

^{1}H NMR (400 MHz, $CDCl_3$)

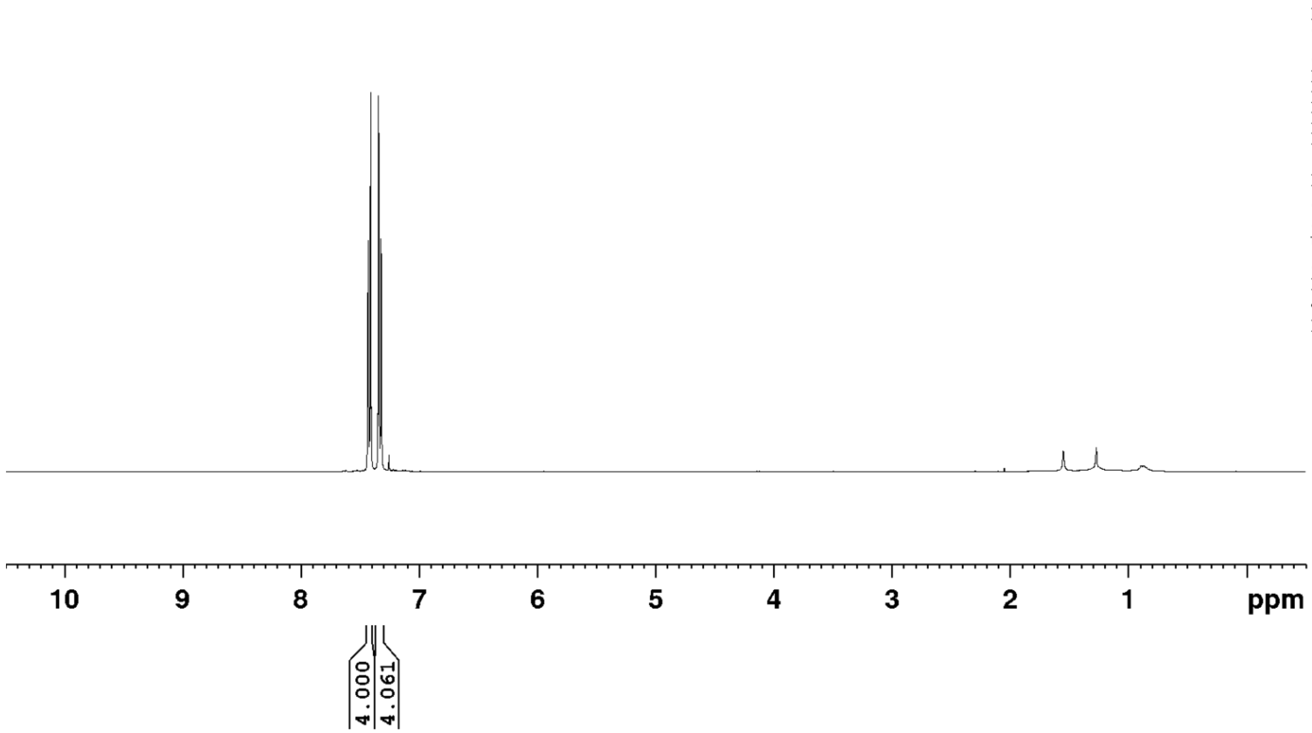

Current Data Parameters
NAME YKC-120-3h 1H
EXPNO 2
PROCNO 1

F2 - Acquisition Parameters
Date_ 20250626
Time 14.58
INSTRUM Avance NEO 400
PROBHD Z104450_0301
PULPROG zg30
TD 32768
SOLVENT CDCl3
NS 16
DS 0
SWH 8196.721
FIDRES 0.500288
AQ 1.9988480
RG 101
DW 0.000
DE 6.50
TE 306.1
D1 1.00000000
TD0 1
SFO1 400.1528010
NUC1 1H
P0 5.00
P1 15.00
PLW1 8.71050000

F2 - Processing parameters
SI 32768
SF 400.1500095 MHz
WDW EM
SSB 0
LB 0.30 Hz
GB 0
PC 1.00

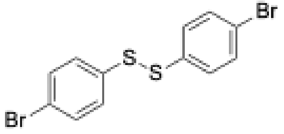

3h

$^{13}C\{^{1}H\}$ NMR (400 MHz, $CDCl_3$)

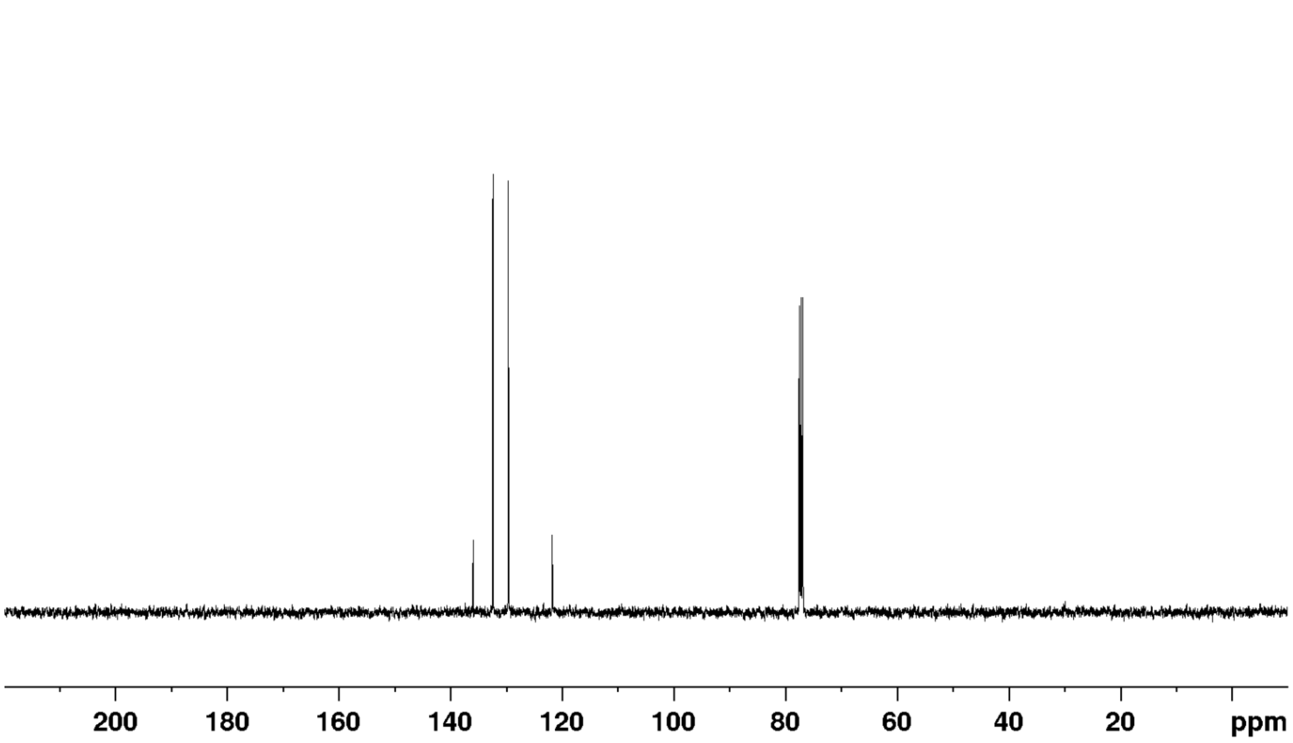


```
Current Data Parameters
NAME                 YKC-120-3h
EXPNO                         3
PROCNO                        1

F2 - Acquisition Parameters
Date_                  20250625
Time                    12.41 h
INSTRUM                   spect
PROBHD      Z108618_0411
PULPROG                  zgpg30
TD                        65536
SOLVENT                   CDCl3
NS                           44
DS                            0
SWH                   28409.092 Hz
FIDRES                 0.866977 Hz
AQ                    1.1534336 sec
RG                       212.49
DW                       17.600 usec
DE                         6.50 usec
TE                        301.5 K
D1                   2.00000000 sec
D11                  0.03000000 sec
TD0                           1
SFO1                100.6258487 MHz
NUC1                        13C
P0                         3.50 usec
P1                        10.50 usec
PLW1                42.50000000 W
SFO2                400.1316005 MHz
NUC2                         1H
CPDPRG[2]               waltz16
PCPD2                     90.00 usec
PLW2                 9.89999962 W
PLW12                0.29363999 W
PLW13                0.14770000 W

F2 - Processing parameters
SI                        32768
SF                  100.6127568 MHz
WDW                          EM
SSB                           0
LB                         3.00 Hz
GB                            0
PC                         1.40
```

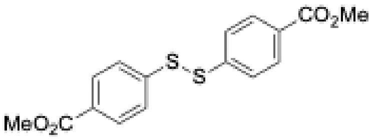

3i

^{1}H NMR (400 MHz, $CDCl_3$)

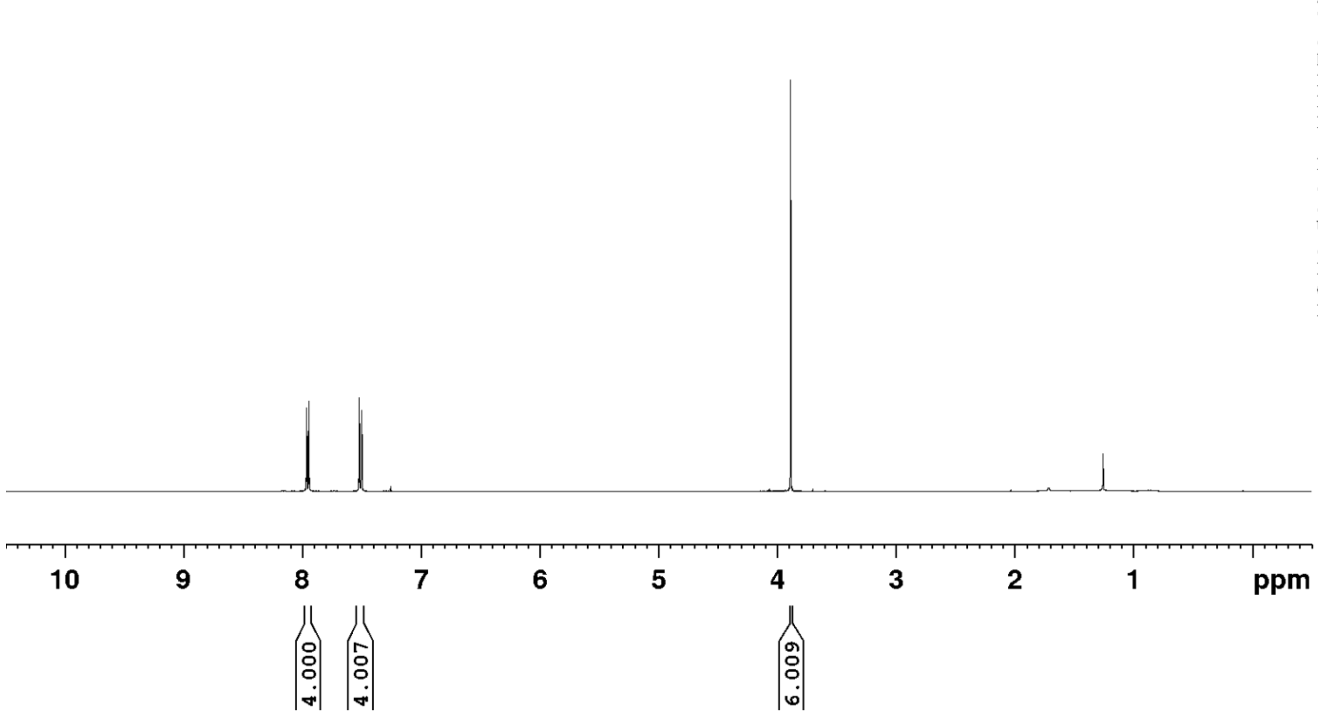


```
Current Data Parameters
NAME                YKC-122-3i
EXPNO                        1
PROCNO                       1

F2 - Acquisition Parameters
Date_                 20250625
Time                   19.31 h
INSTRUM                  spect
PROBHD      Z108618_0411
PULPROG                   zg30
TD                       32768
SOLVENT                  CDCl3
NS                          16
DS                           0
SWH                   8802.817 Hz
FIDRES                0.537281 Hz
AQ                   1.8612224 sec
RG                      118.08
DW                      56.800 usec
DE                       15.08 usec
TE                       301.7 K
D1                  1.00000000 sec
TD0                          1
SFO1               400.1328009 MHz
NUC1                        1H
P0                        5.17 usec
P1                       15.50 usec
PLW1                9.89999962 W

F2 - Processing parameters
SI                      131072
SF                 400.1300093 MHz
WDW                         EM
SSB                          0
LB                           0 Hz
GB                           0
PC                        1.00
```

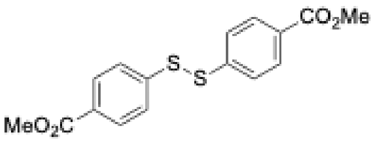

3i

$^{13}C\{^{1}H\}$ NMR (400 MHz, $CDCl_3$)

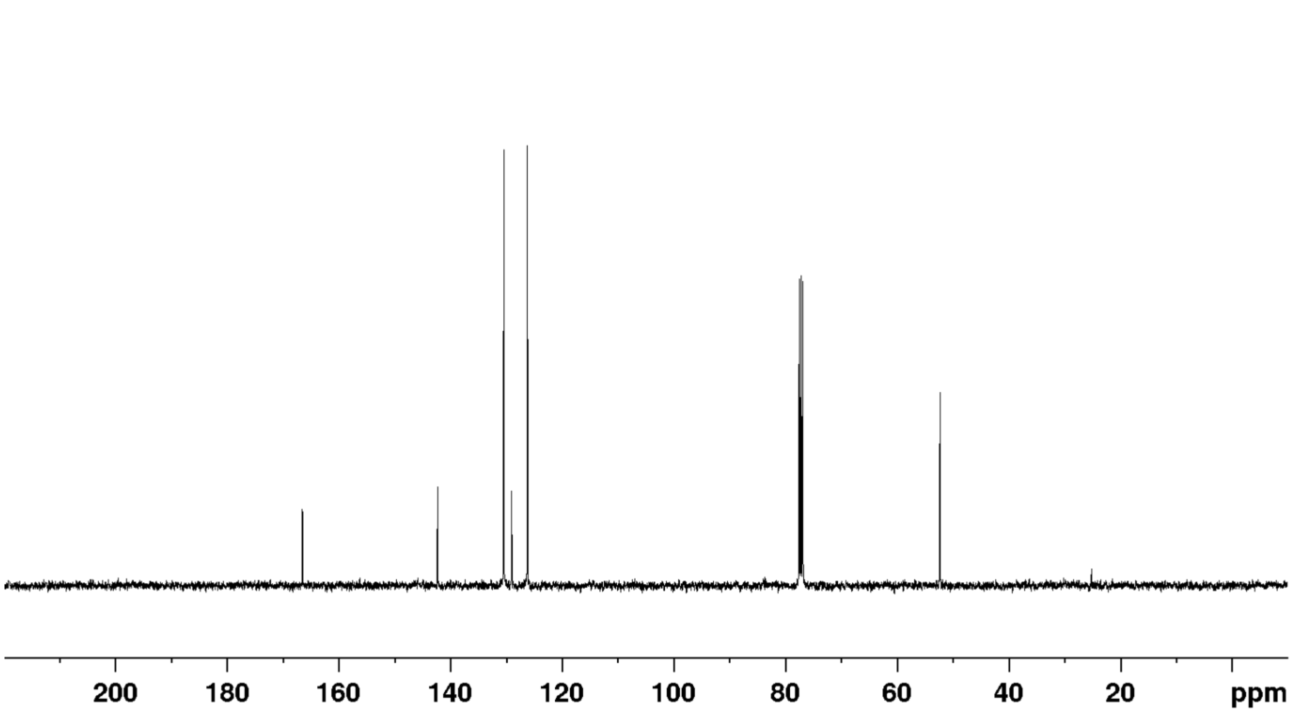

Current Data Parameters
NAME YKC-122-3i
EXPNO 2
PROCNO 1

F2 - Acquisition Parameters
Date_ 20250625
Time 19.35 h
INSTRUM spect
PROBHD Z108618_0411
PULPROG zgpg30
TD 65536
SOLVENT CDCl3
NS 50
DS 0
SWH 28409.092 Hz
FIDRES 0.866977 Hz
AQ 1.1534336 sec
RG 212.49
DW 17.600 usec
DE 6.50 usec
TE 301.7 K
D1 2.00000000 sec
D11 0.03000000 sec
TD0 1
SFO1 100.6258487 MHz
NUC1 13C
P0 3.50 usec
P1 10.50 usec
PLW1 42.50000000 W
SFO2 400.1316005 MHz
NUC2 1H
CPDPRG[2] waltz16
PCPD2 90.00 usec
PLW2 9.89999962 W
PLW12 0.29363999 W
PLW13 0.14770000 W

F2 - Processing parameters
SI 32768
SF 100.6127579 MHz
WDW EM
SSB 0
LB 3.00 Hz
GB 0
PC 1.40

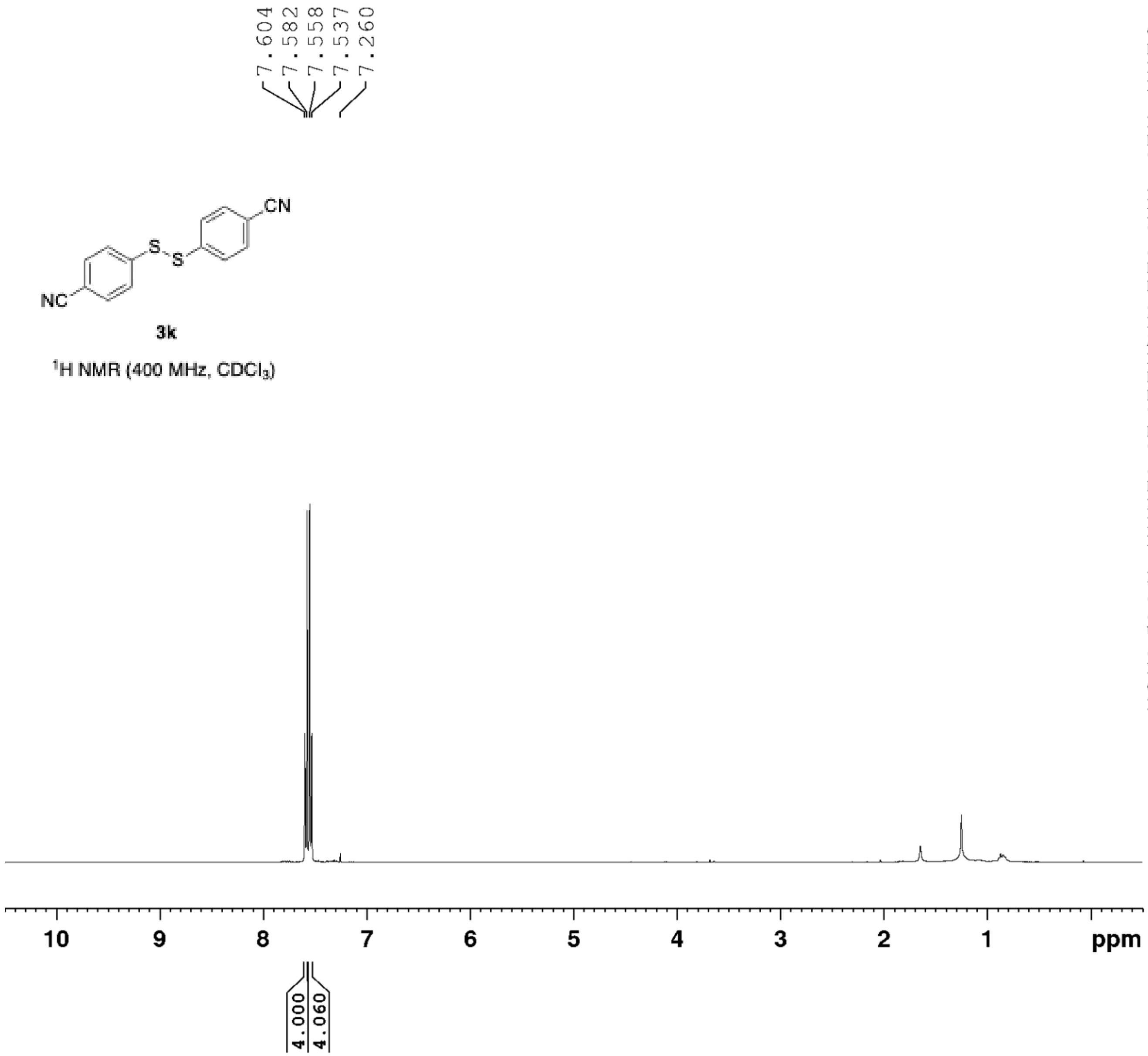

Current Data Parameters
NAME YKC-126-3k
EXPNO 1
PROCNO 1

F2 - Acquisition Parameters
Date_ 20250704
Time 19.03 h
INSTRUM spect
PROBHD Z108618_0411
PULPROG zg30
TD 32768
SOLVENT CDCl3
NS 16
DS 0
SWH 8802.817 Hz
FIDRES 0.537281 Hz
AQ 1.8612224 sec
RG 118.08
DW 56.800 usec
DE 15.08 usec
TE 298.0 K
D1 1.00000000 sec
TD0 1
SFO1 400.1328009 MHz
NUC1 1H
P0 5.17 usec
P1 15.50 usec
PLW1 9.89999962 W

F2 - Processing parameters
SI 131072
SF 400.1300095 MHz
WDW EM
SSB 0
LB 0 Hz
GB 0
PC 1.00

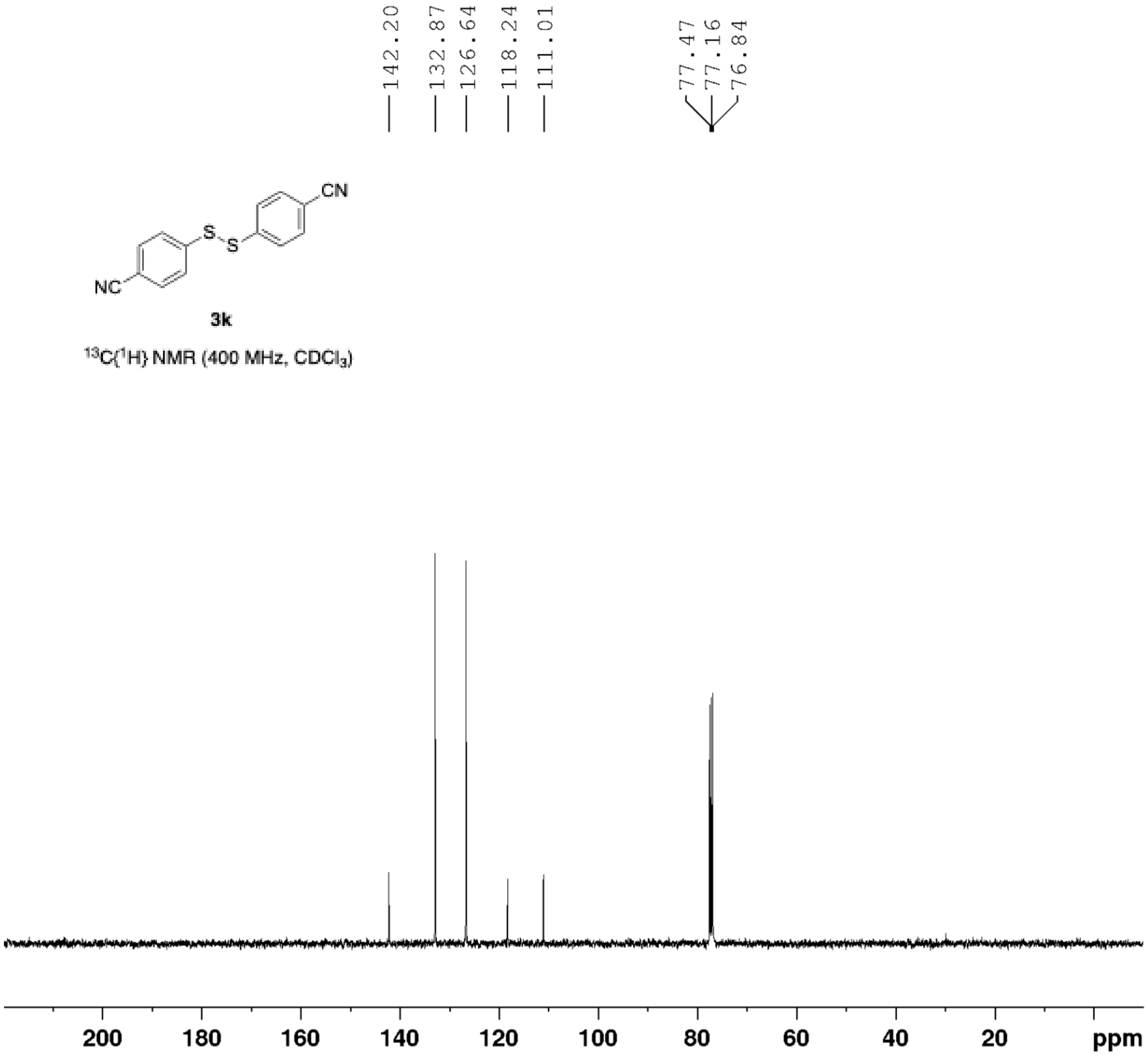

3k

$^{13}C\{^1H\}$ NMR (400 MHz, $CDCl_3$)

Current Data Parameters		
NAME	YKC-126-3k	
EXPNO	2	
PROCNO	1	
F2 - Acquisition Parameters		
Date_	20250704	
Time	19.08	h
INSTRUM	spect	
PROBHD	Z108618_0411	
PULPROG	zgpg30	
TD	65536	
SOLVENT	CDCl3	
NS	46	
DS	0	
SWH	28409.092	Hz
FIDRES	0.866977	Hz
AQ	1.1534336	sec
RG	212.49	
DW	17.600	usec
DE	6.50	usec
TE	298.0	K
D1	2.00000000	sec
D11	0.03000000	sec
TD0	1	
SFO1	100.6258487	MHz
NUC1	13C	
P0	3.50	usec
P1	10.50	usec
PLW1	42.50000000	W
SFO2	400.1316005	MHz
NUC2	1H	
CPDPRG[2]	waltz16	
PCPD2	90.00	usec
PLW2	9.89999962	W
PLW12	0.29363999	W
PLW13	0.14770000	W
F2 - Processing parameters		
SI	32768	
SF	100.6127611	MHz
WDW	EM	
SSB	0	
LB	3.00	Hz
GB	0	
PC	1.40	

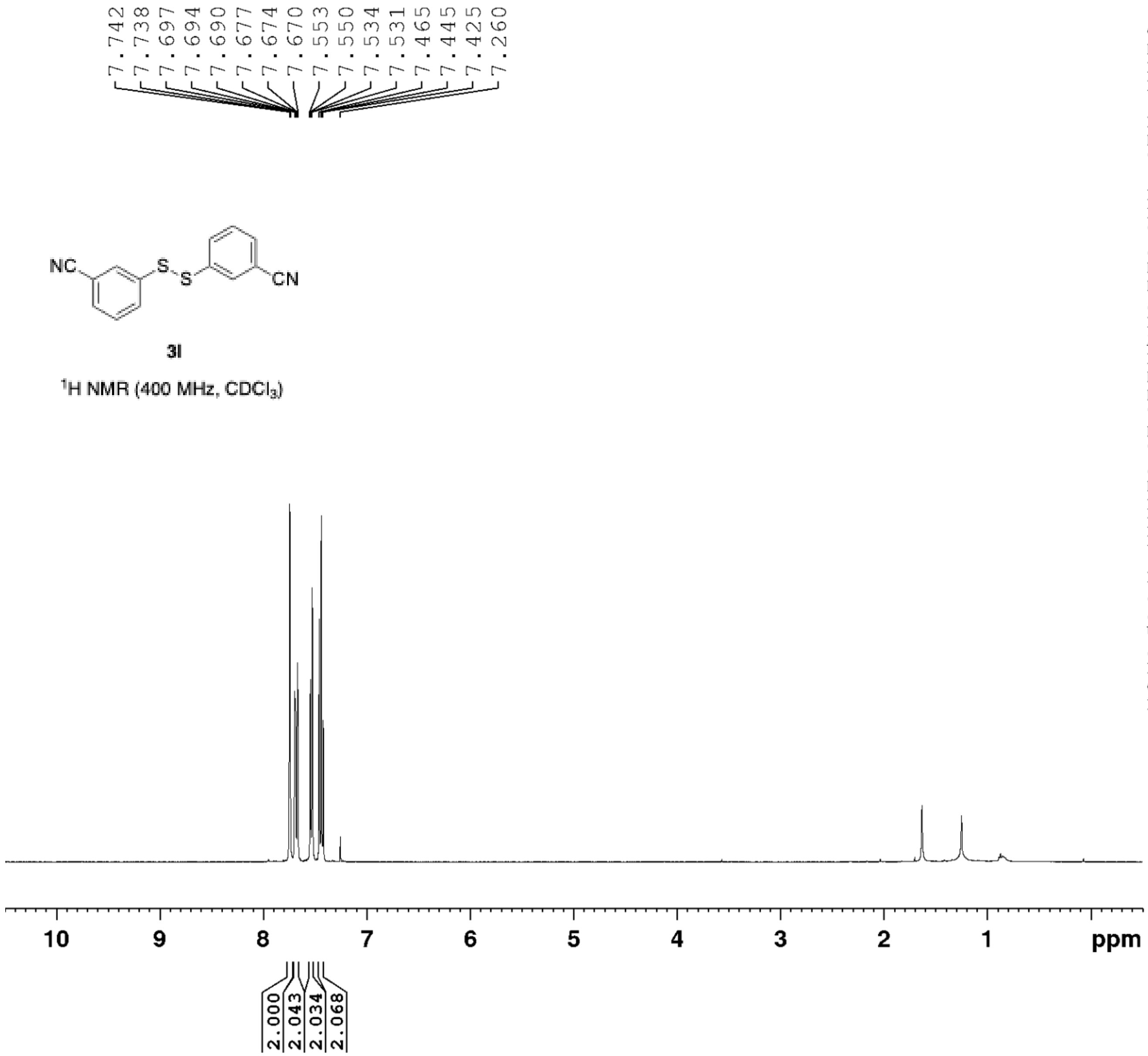

3l

^1H NMR (400 MHz, $CDCl_3$)

Current Data Parameters
NAME YKC-133-31
EXPNO 1
PROCNO 1

F2 - Acquisition Parameters
Date_ 20250716
Time 19.50 h
INSTRUM spect
PROBHD Z108618_0411
PULPROG zg30
TD 32768
SOLVENT CDCl3
NS 16
DS 0
SWH 8802.817 Hz
FIDRES 0.537281 Hz
AQ 1.8612224 sec
RG 133.5
DW 56.800 usec
DE 15.08 usec
TE 298.0 K
D1 1.00000000 sec
TD0 1
SFO1 400.1328009 MHz
NUC1 1H
P0 5.17 usec
P1 15.50 usec
PLW1 9.89999962 W

F2 - Processing parameters
SI 131072
SF 400.1300095 MHz
WDW EM
SSB 0
LB 0 Hz
GB 0
PC 1.00

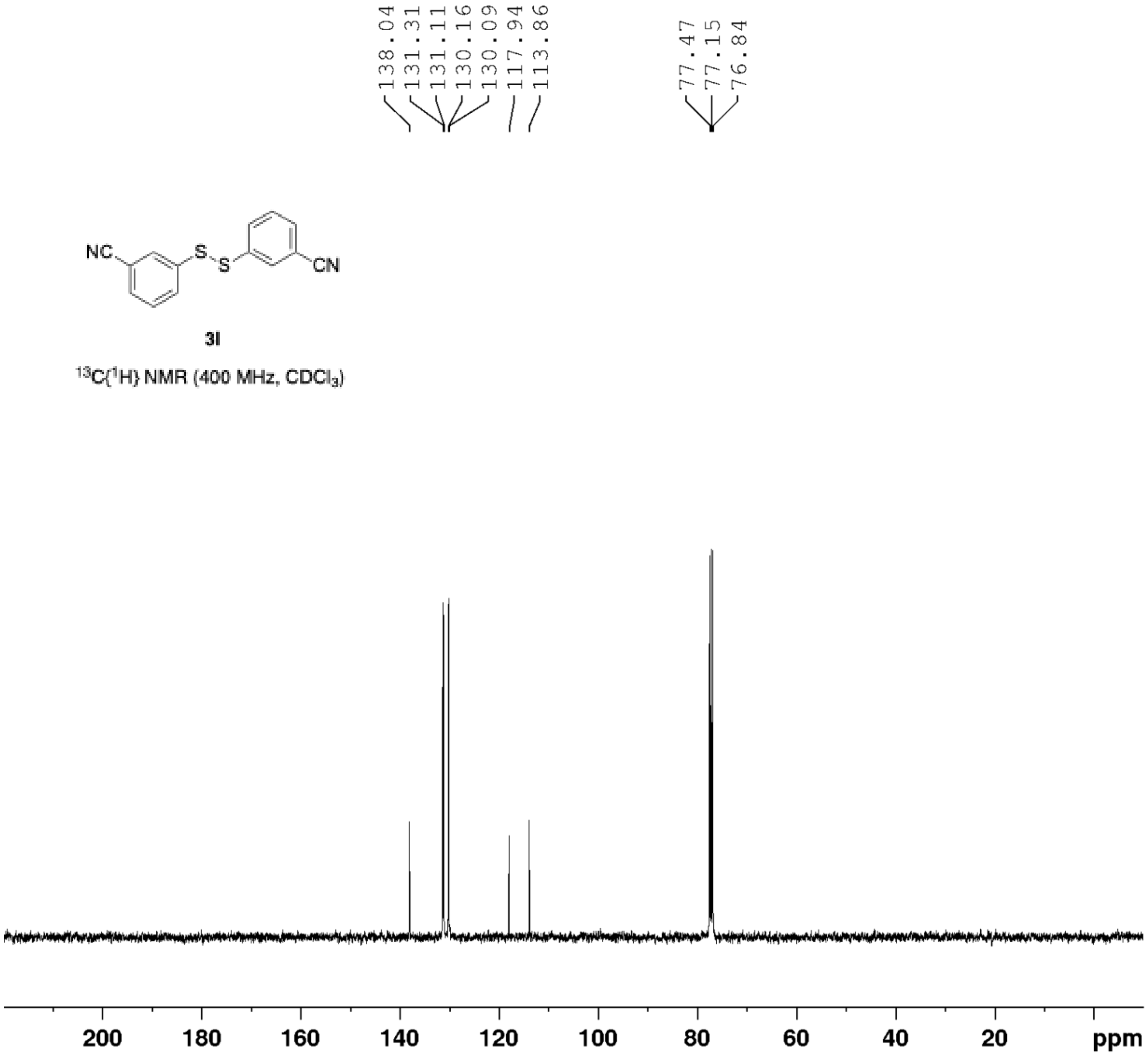

3l

$^{13}C\{^1H\}$ NMR (400 MHz, $CDCl_3$)


```
Current Data Parameters
NAME                YKC-133-31
EXPNO                        2
PROCNO                       1

F2 - Acquisition Parameters
Date_                 20250716
Time                   19.56 h
INSTRUM                  spect
PROBHD     Z108618_0411
PULPROG                 zgpg30
TD                       65536
SOLVENT                  CDCl3
NS                          77
DS                           0
SWH                  28409.092 Hz
FIDRES                0.866977 Hz
AQ                   1.1534336 sec
RG                      212.49
DW                      17.600 usec
DE                        6.50 usec
TE                       298.1 K
D1                  2.00000000 sec
D11                 0.03000000 sec
TD0                          1
SFO1               100.6258487 MHz
NUC1                       13C
P0                        3.50 usec
P1                       10.50 usec
PLW1               42.50000000 W
SFO2               400.1316005 MHz
NUC2                        1H
CPDPRG[2]              waltz16
PCPD2                    90.00 usec
PLW2                9.89999962 W
PLW12               0.29363999 W
PLW13               0.14770000 W

F2 - Processing parameters
SI                       32768
SF                 100.6127611 MHz
WDW                         EM
SSB                          0
LB                        3.00 Hz
GB                           0
PC                        1.40
```

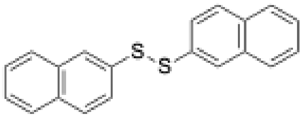

3n

^{1}H NMR (400 MHz, $CDCl_3$)

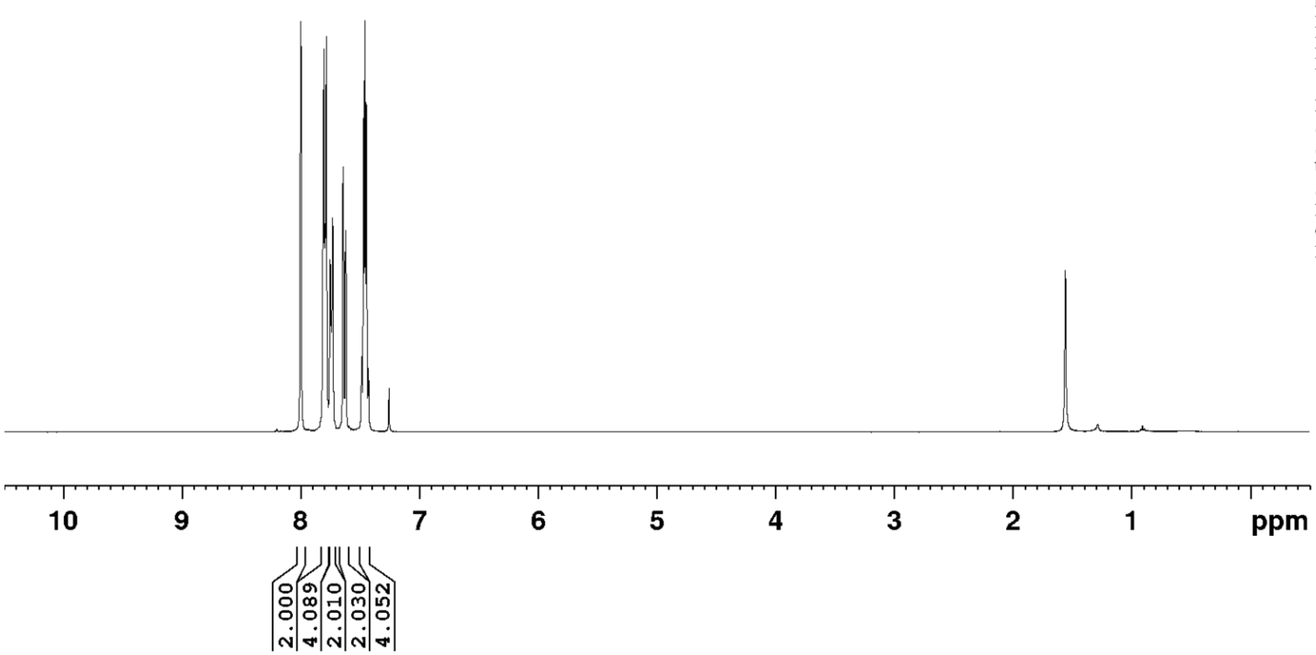


```
Current Data Parameters
NAME                YKC-129-3n
EXPNO                        4
PROCNO                       1

F2 - Acquisition Parameters
Date_                 20250711
Time                   17.56 h
INSTRUM                  spect
PROBHD      Z108618_0411
PULPROG                   zg30
TD                       32768
SOLVENT                  CDCl3
NS                          16
DS                           0
SWH                   8802.817 Hz
FIDRES                0.537281 Hz
AQ                   1.8612224 sec
RG                      143.54
DW                      56.800 usec
DE                       15.08 usec
TE                       298.0 K
D1                  1.00000000 sec
TD0                          1
SFO1               400.1328009 MHz
NUC1                        1H
P0                        5.17 usec
P1                       15.50 usec
PLW1                9.89999962 W

F2 - Processing parameters
SI                      131072
SF                 400.1300095 MHz
WDW                         EM
SSB                          0
LB                           0 Hz
GB                           0
PC                        1.00
```

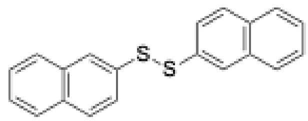

3n

$^{13}C\{^1H\}$ NMR (400 MHz, $CDCl_3$)

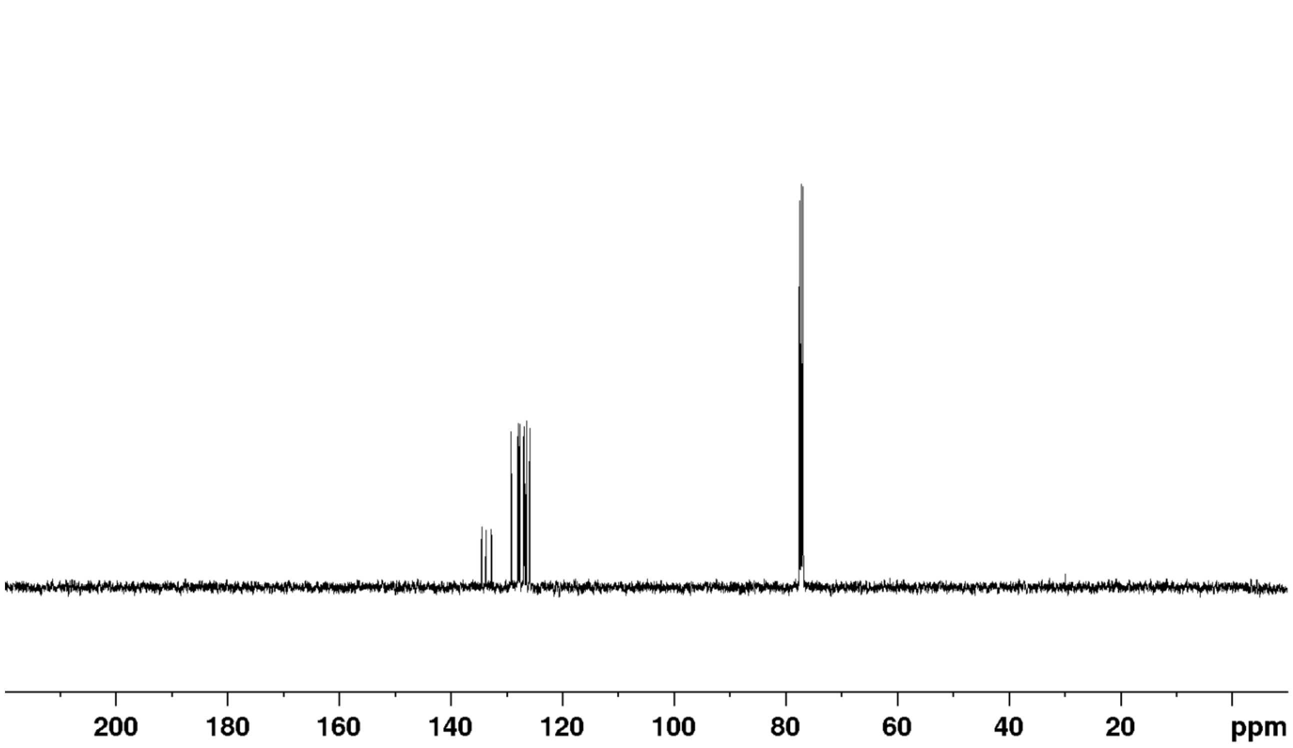


```
Current Data Parameters
NAME                YKC-129-3n
EXPNO                        2
PROCNO                       1

F2 - Acquisition Parameters
Date_                 20250708
Time                     17.37 h
INSTRUM                  spect
PROBHD     Z108618_0411
PULPROG                 zgpg30
TD                       65536
SOLVENT                  CDCl3
NS                          50
DS                           0
SWH                  28409.092 Hz
FIDRES                0.866977 Hz
AQ                   1.1534336 sec
RG                      212.49
DW                      17.600 usec
DE                        6.50 usec
TE                       298.1 K
D1                  2.00000000 sec
D11                 0.03000000 sec
TD0                          1
SFO1               100.6258487 MHz
NUC1                       13C
P0                        3.50 usec
P1                       10.50 usec
PLW1               47.29999924 W
SFO2               400.1316005 MHz
NUC2                        1H
CPDPRG[2]              waltz16
PCPD2                    90.00 usec
PLW2                         0 W
PLW12               0.32330000 W
PLW13               0.16236000 W

F2 - Processing parameters
SI                       32768
SF                 100.6127577 MHz
WDW                         EM
SSB                          0
LB                        3.00 Hz
GB                           0
PC                        1.40
```

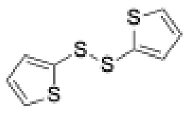

3o

^{1}H NMR (400 MHz, $CDCl_3$)

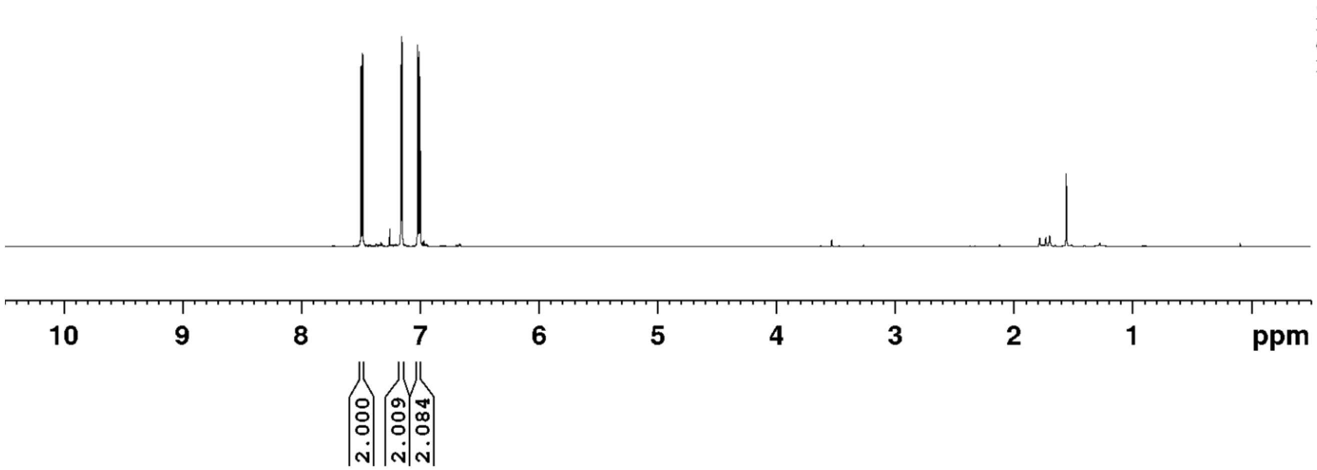


```
Current Data Parameters
NAME                YKC-123-3o
EXPNO                        3
PROCNO                       1

F2 - Acquisition Parameters
Date_                 20250806
Time                     13.38 h
INSTRUM                  spect
PROBHD     Z108618_0411
PULPROG                   zg30
TD                       32768
SOLVENT                  CDCl3
NS                          16
DS                           0
SWH                   8802.817 Hz
FIDRES                0.537281 Hz
AQ                   1.8612224 sec
RG                       133.5
DW                      56.800 usec
DE                       15.08 usec
TE                       298.0 K
D1                  1.00000000 sec
TD0                          1
SFO1               400.1328009 MHz
NUC1                        1H
P0                        5.17 usec
P1                       15.50 usec
PLW1                9.89999962 W

F2 - Processing parameters
SI                      131072
SF                 400.1300096 MHz
WDW                         EM
SSB                          0
LB                           0 Hz
GB                           0
PC                        1.00
```

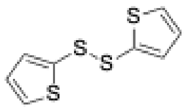

3o

$^{13}C\{^{1}H\}$ NMR (400 MHz, $CDCl_3$)

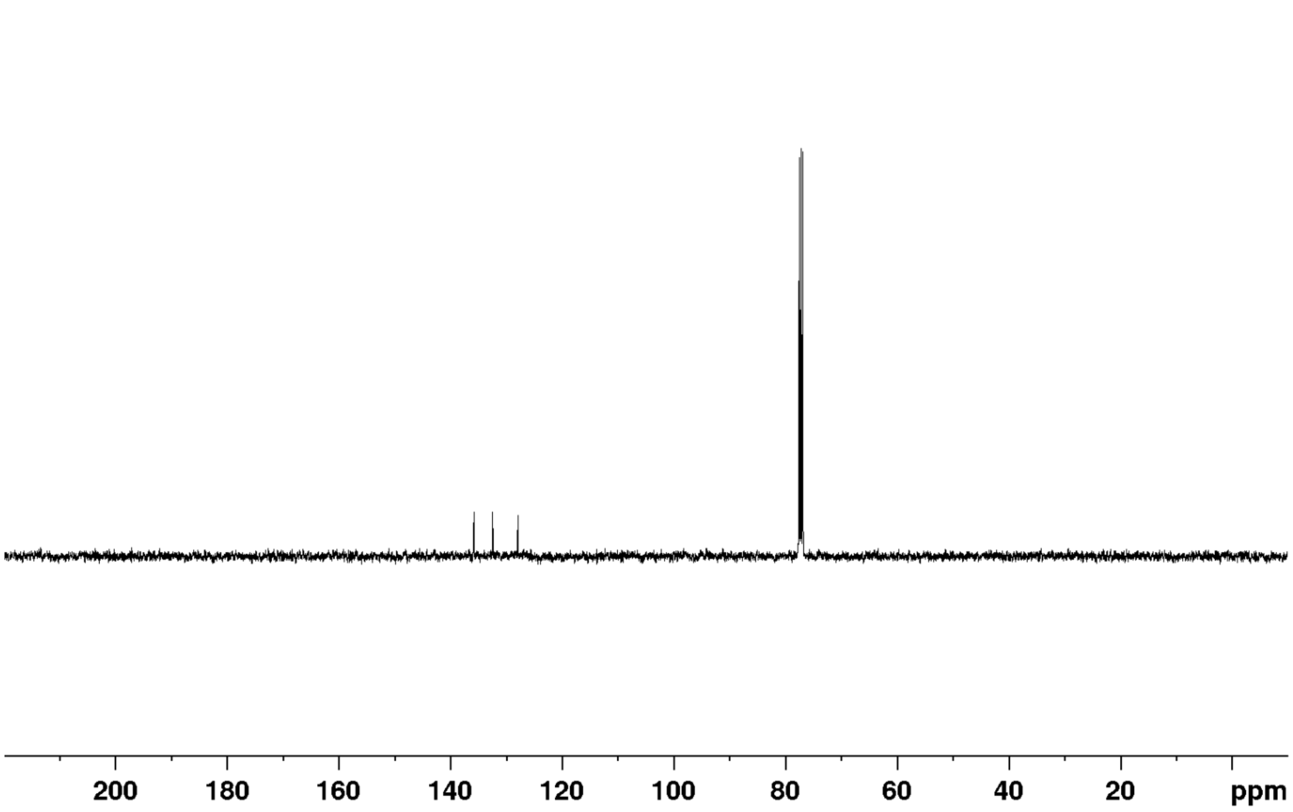


```
Current Data Parameters
NAME                YKC-123-3o
EXPNO                        2
PROCNO                       1

F2 - Acquisition Parameters
Date_                 20250801
Time                     14.18 h
INSTRUM                  spect
PROBHD     Z108618_0411
PULPROG                zgpg30
TD                       65536
SOLVENT                  CDCl3
NS                          63
DS                           0
SWH                  28409.092 Hz
FIDRES                0.866977 Hz
AQ                   1.1534336 sec
RG                      212.49
DW                      17.600 usec
DE                        6.50 usec
TE                       298.1 K
D1                  2.00000000 sec
D11                 0.03000000 sec
TD0                          1
SFO1               100.6258487 MHz
NUC1                       13C
P0                        3.50 usec
P1                       10.50 usec
PLW1               42.50000000 W
SFO2               400.1316005 MHz
NUC2                        1H
CPDPRG[2]              waltz16
PCPD2                    90.00 usec
PLW2                9.89999962 W
PLW12               0.29363999 W
PLW13               0.14770000 W

F2 - Processing parameters
SI                       32768
SF                 100.6127544 MHz
WDW                         EM
SSB                          0
LB                        3.00 Hz
GB                           0
PC                        1.40
```

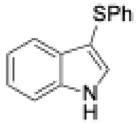

7

^{1}H NMR (400 MHz, $CDCl_3$)

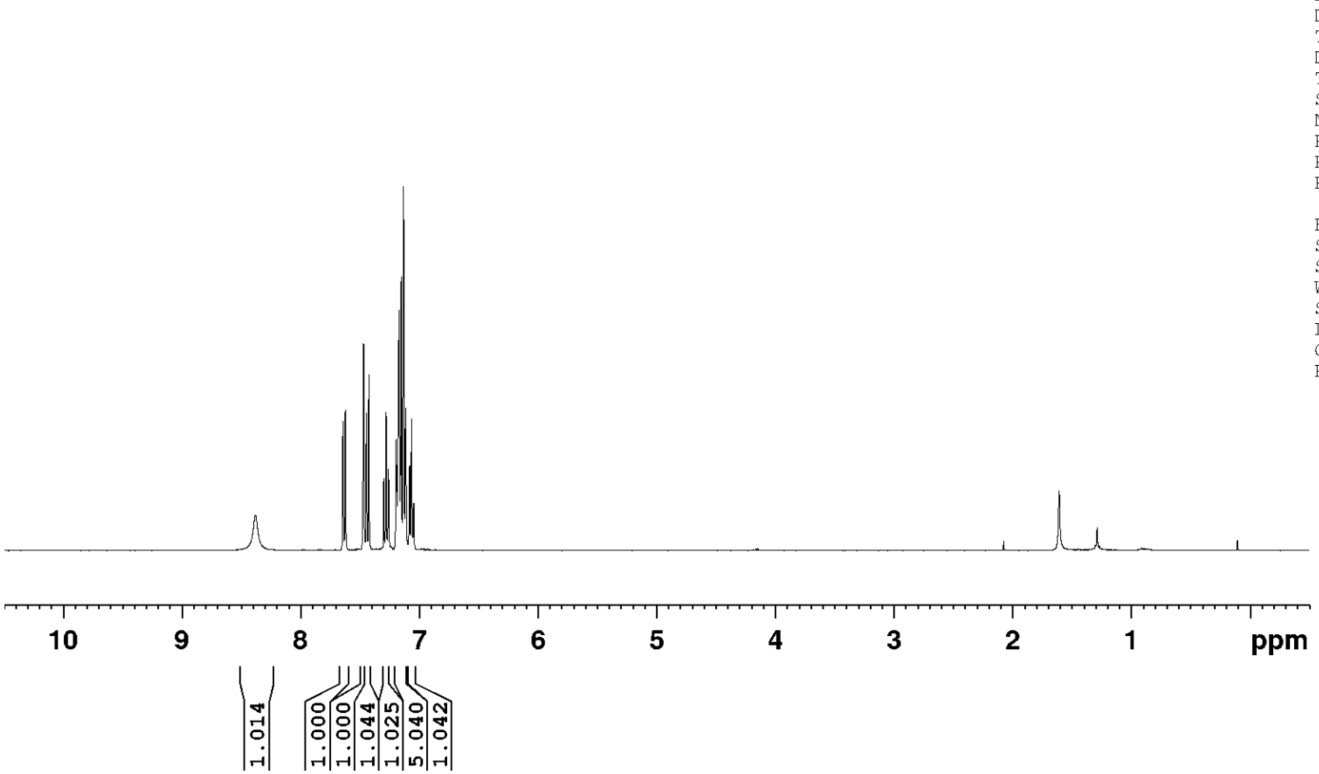


```
Current Data Parameters
NAME              YKC-154..2 7
EXPNO                        1
PROCNO                       1

F2 - Acquisition Parameters
Date_                 20251021
Time                     17.37 h
INSTRUM                  spect
PROBHD      Z108618_0411
PULPROG                   zg30
TD                       32768
SOLVENT                  CDCl3
NS                          16
DS                           0
SWH                   8802.817 Hz
FIDRES                0.537281 Hz
AQ                   1.8612224 sec
RG                      143.54
DW                      56.800 usec
DE                       15.08 usec
TE                       298.1 K
D1                  1.00000000 sec
TD0                          1
SFO1               400.1328009 MHz
NUC1                        1H
P0                        5.17 usec
P1                       15.50 usec
PLW1                9.89999962 W

F2 - Processing parameters
SI                      131072
SF                 400.1300098 MHz
WDW                         EM
SSB                          0
LB                           0 Hz
GB                           0
PC                        1.00
```

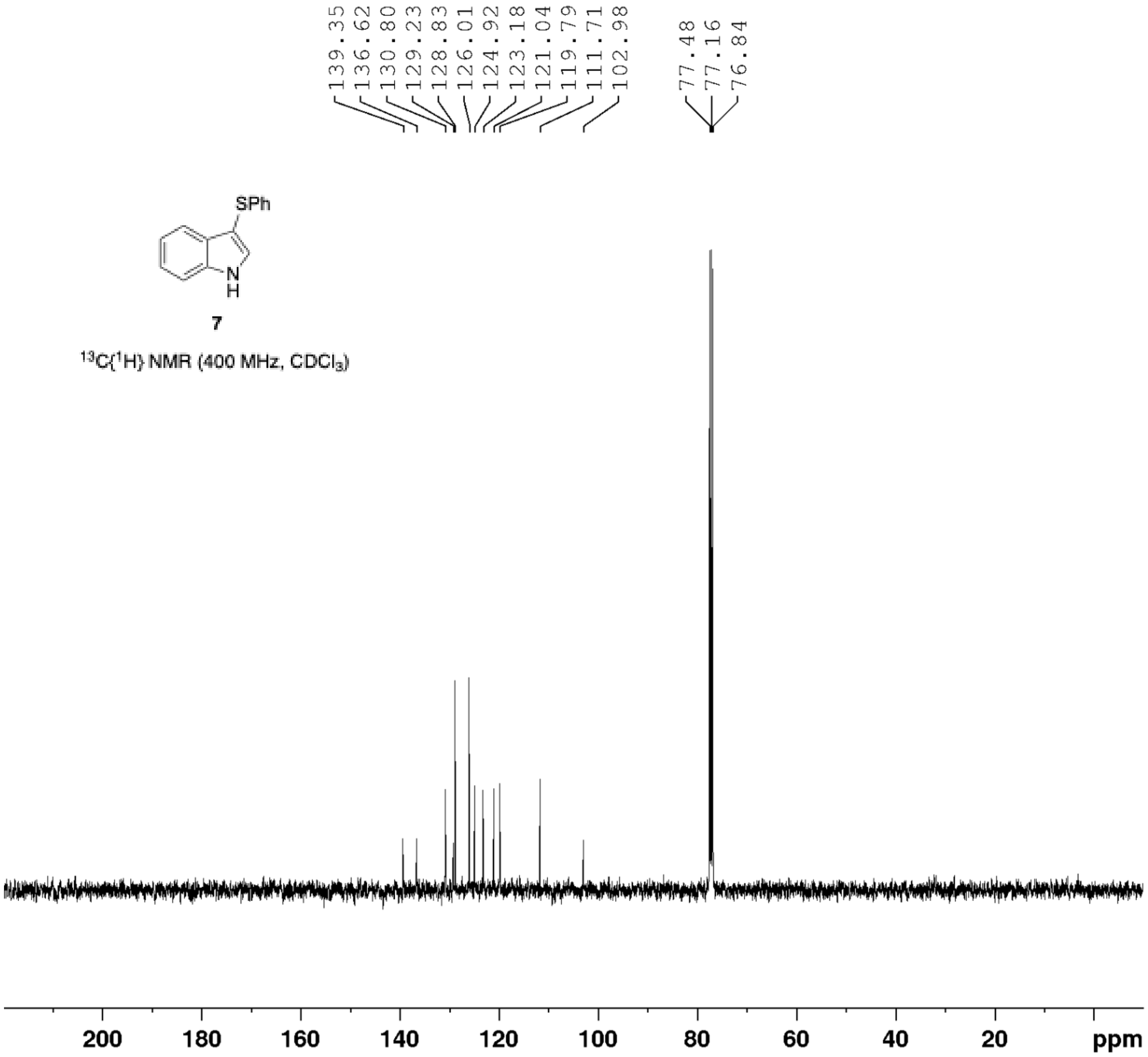

Current Data Parameters

NAME	YKC-154..2 7	
EXPNO	2	
PROCNO	1	

F2 - Acquisition Parameters

Date_	20251021	
Time	17.42	h
INSTRUM	spect	
PROBHD	Z108618_0411	
PULPROG	zgpg30	
TD	65536	
SOLVENT	CDCl3	
NS	59	
DS	0	
SWH	28409.092	Hz
FIDRES	0.866977	Hz
AQ	1.1534336	sec
RG	212.49	
DW	17.600	usec
DE	6.50	usec
TE	298.1	K
D1	2.00000000	sec
D11	0.03000000	sec
TD0	1	
SFO1	100.6258487	MHz
NUC1	13C	
P0	3.50	usec
P1	10.50	usec
PLW1	42.50000000	W
SFO2	400.1316005	MHz
NUC2	1H	
CPDPRG[2]	waltz16	
PCPD2	90.00	usec
PLW2	9.89999962	W
PLW12	0.29363999	W
PLW13	0.14770000	W

F2 - Processing parameters

SI	32768	
SF	100.6127588	MHz
WDW	EM	
SSB	0	
LB	3.00	Hz
GB	0	
PC	1.40	
